# Casein kinase 1γ acts as a molecular switch for cell polarization through phosphorylation of the polarity factor Tea1 in fission yeast

**DOI:** 10.1111/gtc.12309

**Published:** 2015-11-02

**Authors:** Takayuki Koyano, Karin Barnouin, Ambrosius P. Snijders, Kazunori Kume, Dai Hirata, Takashi Toda

**Affiliations:** ^1^ Lincoln's Inn Fields LaboratoryThe Francis Crick Institute44 Lincoln's Inn FieldsLondonWC2A 3LYUK; ^2^ Clare Hall LaboratoryThe Francis Crick InstituteBlanche LaneSouth MimmsPotters BarHertfordshireEN6 3LDUK; ^3^ Department of Molecular BiotechnologyGraduate School of Advanced Sciences of MatterHiroshima University1‐3‐1 KagamiyamaHigashi‐Hiroshima739‐8530Japan; ^4^ Division of Molecular GeneticsShigei Medical Research Institute2117 YamadaOkayama701‐0202Japan; ^5^ Asahi‐Shuzo Sake Brewing Co., Ltd880‐1 AsahiNagaoka949‐5494Japan; ^6^ Hiroshima Research Center for Healthy Aging (HiHA)Department of Molecular BiotechnologyGraduate School of Advanced Sciences of MatterHiroshima University1‐3‐1 KagamiyamaHigashi‐Hiroshima739‐8530Japan

## Abstract

Fission yeast undergoes growth polarity transition from monopolar to bipolar during G2 phase, designated NETO (New End Take Off). It is known that NETO onset involves two prerequisites, the completion of DNA replication and attainment of a certain cell size. However, the molecular mechanism remains unexplored. Here, we show that casein kinase 1γ, Cki3 is a critical determinant of NETO onset. Not only did *cki3*∆ cells undergo NETO during G1‐ or S‐phase, but they also displayed premature NETO under unperturbed conditions with a smaller cell size, leading to cell integrity defects. Cki3 interacted with the polarity factor Tea1, of which phosphorylation was dependent on Cki3 kinase activity. GFP nanotrap of Tea1 by Cki3 led to Tea1 hyperphosphorylation with monopolar growth, whereas the same entrapment by kinase‐dead Cki3 resulted in converse bipolar growth. Intriguingly, the Tea1 interactor Tea4 was dissociated from Tea1 by Cki3 entrapment. Mass spectrometry identified four phosphoserine residues within Tea1 that were hypophosphorylated in *cki3*∆ cells. Phosphomimetic Tea1 mutants showed compromised binding to Tea4 and NETO defects, indicating that these serine residues are critical for protein–protein interaction and NETO onset. Our findings provide significant insight into the mechanism by which cell polarization is regulated in a spatiotemporal manner.

## Introduction

Cell polarization is of fundamental importance for many biological processes. These include asymmetric cell growth leading to proper cell morphogenesis, polarized cell migration, and mitotic spindle positioning that is required for asymmetric cell division and differentiation (McCaffrey & Macara 2009). In principle, cell polarity can be established by accumulation of ‘polarity factors’, consisting of a cohort of proteins involved in the establishment and maintenance of cell polarity, at a specific cellular site in response to internal and external cues.

The fission yeast *Schizosaccharomyces pombe* offers an ideal system in which to study the molecular pathways underlying cell polarization. These rod‐shaped cells are highly polarized; cells grow only from cell tips with constant width. Immediately after medial cell division, cells start to grow in a monopolar manner by activating the ‘old end’, which already existed before cell division. Subsequently, at some point during G2 phase of the cell cycle, cells undergo a drastic polarity transition from monopolar to bipolar growth. This regulatory point is referred to as NETO (New End Take Off), in which the ‘new end’ that was produced by cell division is now activated (Mitchison & Nurse 1985). For NETO to take place, two requirements must be fulfilled: DNA replication and attainment of a certain cell size. However, the detailed mechanisms by which the timing of NETO onset is regulated remain largely unknown. A number of monopolar mutants with defects in NETO have been described, and the complex molecular network has started to emerge (Huisman & Brunner 2011). Nonetheless, genes whose mutations display premature NETO under unperturbed conditions, which should be instrumental in deciphering the regulatory mechanism, have not been identified.

The microtubule and actin cytoskeletons play a pivotal role in establishment and maintenance of cell morphology in fission yeast as in other eukaryotes (Chang & Martin 2009). During interphase, antiparallel microtubules are organized along the cell axis and become nucleated from microtubule organizing centers, which are situated around the nucleus. Microtubules serve to position the nucleus in the cell middle and deliver a group of polarity factors to the cell ends, thereby activating actin/formin‐dependent cell growth.

The kelch‐repeat protein Tea1 and the SH3‐ and protein phosphatase 1 (PP1)‐binding domain‐containing Tea4 play a central role in the control of growth polarity control. These two proteins form a complex and are delivered to the cell tips through microtubules (Mata & Nurse 1997; Martin *et al*. 2005; Tatebe *et al*. 2005; Alvarez‐Tabares *et al*. 2007). Once transported to the tips, the complex is tethered to the membrane through the prenylated anchor Mod5 (Snaith & Sawin 2003). In the absence of Tea1 or Tea4, cells are incapable of executing NETO: these cells display monopolar growth often with aberrant bent/branched morphologies (Mata & Nurse 1997; Martin *et al*. 2005; Tatebe *et al*. 2005).

Conserved casein kinase 1 (CK1) comprises a large protein family, consisting of four isoforms (α, γ, δ and ε) and serve a wide range of cellular functions as key signaling molecules (Knippschild *et al*. 2014). CK1γ is localized to the plasma membrane depending on the C‐terminal double‐cysteine motif, which is modified by palmitoylation (Davidson *et al*. 2005). We previously showed that fission yeast CK1γ, known as Cki3, is localized to the plasma membrane and that Cki3 kinase activity is required for the delay in NETO onset downstream of Cds1/CHK2 and calcineurin when S‐phase is blocked (Kume *et al*. 2011; Koyano *et al*. 2015).

In this present study, we found that Cki3 also played a critical role in the determination of the NETO timing during an unperturbed cell cycle. Further analysis showed that Cki3 acted through Tea1, which was negatively regulated by Cki3‐mediated phosphorylation. We discuss herein how NETO onset is regulated in a spatiotemporal manner through Cki3 and Tea1.

## Results

### Cki3 determines the timing of NETO during an unperturbed cell cycle and ensures cell integrity

**Figure 1:**
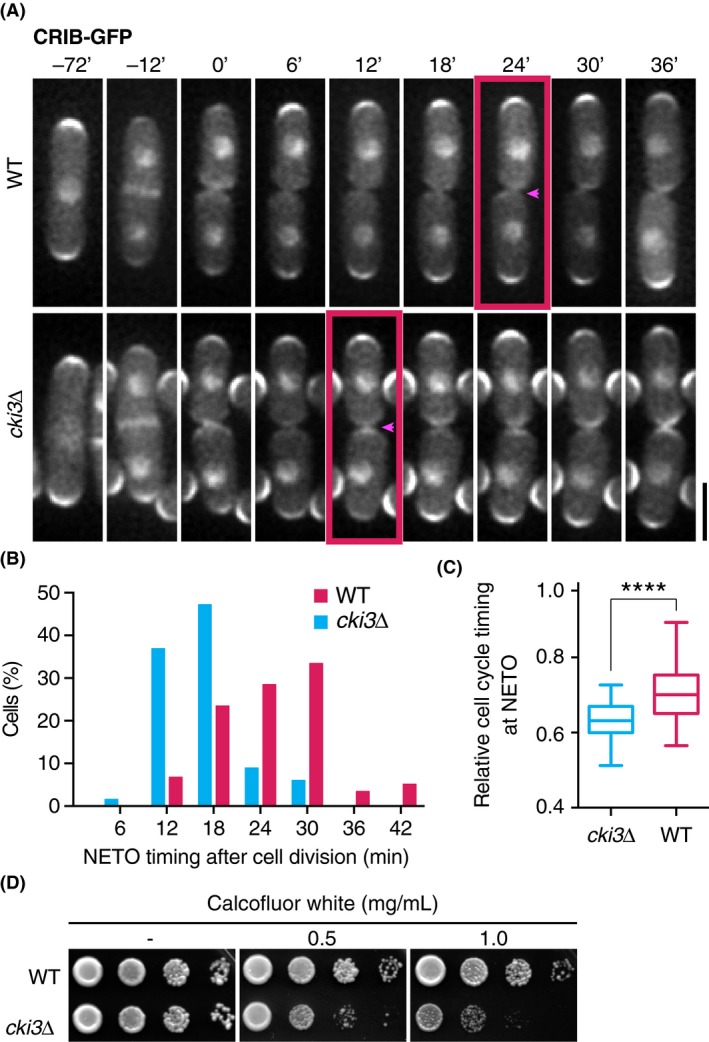
*cki3*∆ cells advanced NETO. (A) Time‐lapse images of CRIB‐GFP in WT and *cki3*∆ cells. Time 0 (min) indicates cell division. Arrowheads denote the initial appearance of CRIB‐GFP signals to new ends. Scale bar, 5 μm. (B) Distribution of the timing of NETO onset. At least 100 cells were observed in WT (red) and *cki3*∆ cells (blue). (C) Relative cell cycle timing of NETO onset. In each cell, cell length at the beginning of NETO was divided by that at cell division (WT;* n* = 51, *cki3*∆; *n* = 41). The box‐and‐whisker plot indicates the minimum and maximum values, the 25th and 75th percentiles, and the median. ****P < 0.0001. (D) Ten‐fold serially diluted WT (top) or *cki3*∆ (bottom) cells were spotted on YE5S in the presence or absence of Calcofluor White and incubated at 36 °C for 2 days.

We sought to examine the role of Cki3 in NETO regulation under unperturbed conditions. To precisely follow polarized growth of individual cells, we introduced the CRIB‐GFP (GFP‐tagged Cdc42/Rac interactive binding domain) reporter (Tatebe *et al*. 2008), which interacts with GTP‐bound active Cdc42, into fission yeast cells. *cki3*∆ cells initiated NETO in the temperature‐sensitive G1 arrest *cdc10‐129* or S‐phase arrest *pol1‐1546* mutant at the restrictive temperature (Fig. S1A,B in Supporting Information), indicating that Cki3 is necessary for inhibiting NETO before the completion of DNA replication as previously shown (Koyano *et al*. 2010, 2015). Inspection of growth patterns by live imaging of exponentially growing wild‐type (WT) cells showed that they underwent NETO on average 25 min after cell division, corresponding to ~0.35 of the cell cycle (scale of 0–1, 0 being set at septation) as previously reported (Mitchison & Nurse 1985; Fig. 1A, top and Fig. 1B,C). In contrast, *cki3*∆ cells initiated NETO earlier, ~17 min after cell division corresponding to ~0.31 of the cell cycle (Fig. 1A, bottom and Fig. 1B,C). Direct observation of growth patterns with conventional bright‐field light microscopy also confirmed earlier onset of NETO in *cki3*∆ cells (Fig. S1C,D in Supporting Information). Consistent with this notion, synchronous culture analysis with centrifugal elutriation confirmed premature NETO onset of *cki3*∆ cells; approximately 40% of small G2 cells at time 0 had already completed NETO (Fig. S1E in Supporting Information). Hence, *cki3*∆ cells showed advanced onset of NETO under an unperturbed cell cycle.

After cell division, unlike old ends, freshly created new ends need to remodel intact cell tips. This process is deemed to be under spatial and temporal control of coordinated biosynthesis of the plasma membrane and cell wall (Estravis *et al*. 2012). We surmised that premature polarized growth at new ends might interfere with this coordination. Therefore, we addressed this proposition by examining sensitivity to a cell wall inhibitor Calcofluor White. As shown in Fig. 1D, *cki3*∆ cells became hypersensitive to this drug, indicating that NETO execution at a proper cell cycle stage was important for the maintenance of cell integrity, although the involvement of Cki3 in cell integrity independent of NETO regulation is also possible. Taken together, we concluded that Cki3 ensured the proper timing of NETO during the cell cycle, which is necessary for a temporal coupling between polarized growth and cell wall organization.

### Phosphorylation of Tea1 is dependent on Cki3

**Figure 2:**
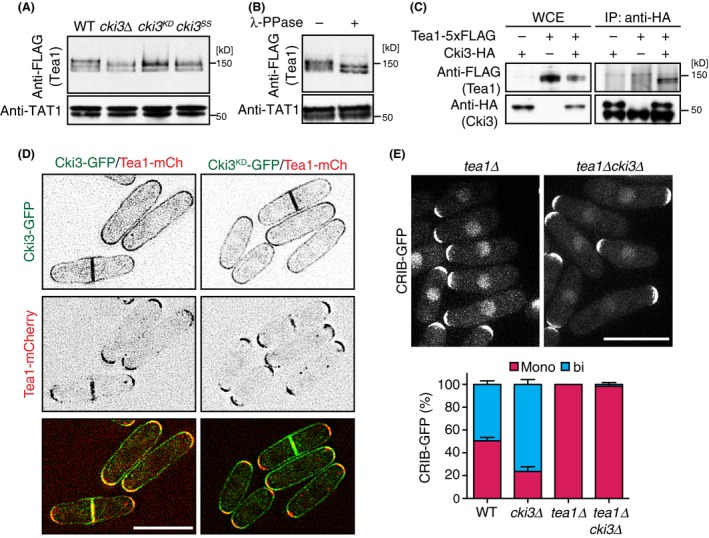
Tea1 phosphorylation is dependent on Cki3. (A) Whole cell extracts were prepared from the indicated strains and immunoblotting carried out with anti‐FLAG and anti‐α‐tubulin antibodies. The positions of molecular weight markers are shown on the right. (B) Extracts prepared from WT cells used in A were incubated in the presence (+) or absence (−) of λ‐phosphatase (PPase). (C) Whole cell extracts were prepared from individual cells and immunoprecipitation carried out with anti‐HA antibody. Immunoprecipitates were immunoblotted with anti‐FLAG and anti‐HA antibodies. (D) Cells expressing Tea1‐mCherry and Cki3‐GFP or Cki3^KD^‐GFP were observed under a fluorescence microscope. The middle section of images is shown. (E) Growth polarity was determined by localization of CRIB‐GFP in individual cells. The top panel shows representative images, and the bottom panel indicates quantification (*n* > 200). Scale bars, 10 μm.

To search for the regulatory factors involved in NETO regulation downstream of Cki3, we first carried out immunoblotting against several known polarity factors on SDS‐PAGE gels and compared side‐by‐side the differences in the mobility of each protein between WT and *cki3*∆ cells. Among the six proteins examined, which included Tea1, Tea3, Tea4, Pom1, Rga4, and Gef1 (Chang & Martin 2009; Huisman & Brunner 2011), we found that Tea1 displayed a faster motility in *cki3*∆ cells than in the WT ones (Fig. 2A and Fig. S2A–C in Supporting Information). Cells containing a kinase‐dead version of Cki3 (Cki3^KD^) or membrane localization‐defective Cki3^SS^ mutant (Koyano *et al*. 2015) also displayed a similar faster mobility of Tea1 (Fig. 2A). Treatment with λ phosphatase (λ‐PPase) confirmed that Tea1 was a phosphoprotein as previously shown (Kim *et al*. 2003); Tea1 mobility was very similar, if not identical, between *cki3*∆ and *cki3* ^*KD*^ */cki3* ^*SS*^ mutant cells (Fig. 2A,B).

Co‐immunoprecipitation experiments showed that Tea1 interacted with Cki3 (Fig. 2C), which is in line with previously reported mass spectrometry data (Snaith *et al*. 2011). In contrast, we did not see any interaction between Cki3 and Tea4, a Tea1 interactor (Martin *et al*. 2005; Tatebe *et al*. 2005), although Tea4 bound PP1, as previously reported (Alvarez‐Tabares *et al*. 2007; Fig. S2D in Supporting Information). Inspection of Cki3‐GFP and Tea1‐mCherry localization in a single cell showed that Tea1 colocalized with Cki3 at the cell tip (Fig. 2D, left), although Cki3 was localized to the whole plasma membrane but with more concentrated signals at the cell tip, as previously shown (Koyano *et al*. 2015). Tea1 localization was not altered in the Cki3^KD^ background (Fig. 2D, right). It is known that Tea1 is required for NETO execution, as *tea1*∆ cells display NETO‐defective monopolar growth (Mata & Nurse 1997). To establish genetic epistasis, we examined the growth patterns of *tea1*∆*cki3*∆ cells. Double mutants displayed monopolar growth patterns, identical to those of the *tea1*∆ single mutant (Fig. 2E). Therefore, *tea1* was epistatic to *cki3* with regard to NETO regulation. In sum, Tea1 is phosphorylated through Cki3 probably at the cell tip, the failure of which leads to premature NETO execution.

### Induced entrapment of Tea1 by Cki3 results in constitutive hyperphosphorylation of Tea1 with monopolar growth

**Figure 3:**
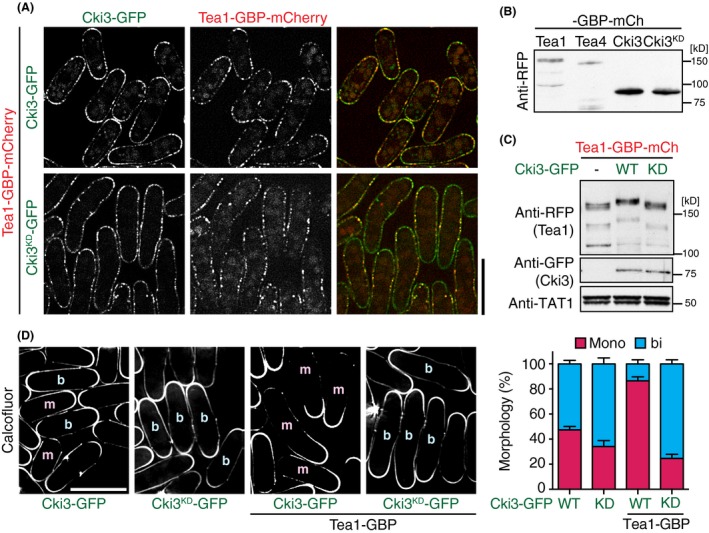
Entrapment of Tea1 by Cki3 leads to Tea1 hyperphosphorylation and inhibits NETO. (A) Cells producing Tea1‐GBP‐mCherry and Cki3‐GFP (top) or Cki3^KD^‐GFP (bottom) were observed, and fluorescence images were taken. (B) Whole cell extracts were prepared from individual strains and immunoblotting carried out with anti‐RFP antibody. (C) Whole cell extracts were prepared from the indicated cells containing Tea1‐GBP‐mCherry and Cki3‐GFP (WT) or Cki3^KD^‐GFP (KD) or Tea1‐GBP‐mCherry only (−), and immunoblotting carried out with the indicated antibodies. (D) Growth polarity of individual cells was determined with Calcofluor White staining. ‘m’ and ‘b’ indicate monopolar and bipolar cells, respectively. Quantification data are shown in the right‐hand side panel.

To address the impact of Tea1 phosphorylation carried out through Cki3 in NETO regulation, we sought to create an artificial situation in which Cki3 could constitutively phosphorylate Tea1. To this end, we implemented the GFP entrapment strategy using the GFP‐binding protein (GBP; Rothbauer *et al*. 2008). Strains containing Tea1 tagged with GBP‐mCherry and Cki3‐GFP or Cki3^KD^‐GFP, all produced from the endogenous promoters, were constructed. Under this entrapment condition, Tea1 was tethered to Cki3 or Cki3^KD^, thereby being localized to the whole plasma membrane (Fig. 3A). Protein levels of wild‐type Cki3 and Cki3^KD^ were comparable and more abundant than those of Tea1 or Tea4 (Fig. 3B). Intriguingly, immunoblotting of Tea1‐GBP‐mCherry in cells containing Cki3‐GFP showed that Tea1 entrapped by wild‐type Cki3‐GFP, but not by Cki3^KD^‐GFP, was hyperphosphorylated (Fig. 3C).

Remarkably, the majority (>85%) of cells containing Cki3‐GFP and Tea1‐GBP‐mCherry showed monopolar growth (Fig. 3D). In stark contrast, cells containing Cki3^KD^‐GFP and Tea1‐GBP‐mCherry displayed, like cells producing only Cki3‐GFP^KD^, mainly (~80%) bipolar growth instead. As Tea1 became colocalized with either wild‐type Cki3 or Cki3^KD^ to the plasma membrane, it was Cki3‐dependent kinase activity, not its subcellular location, which was responsible for opposing growth polarities. Of note, the cell length of entrapment strains was somewhat longer than that of the WT cells, with some cells showing irregular shapes (in particular cells containing Cki3^KD^‐GFP and Tea1‐GBP‐mCherry), the reason for which is currently not yet being investigated further. It should, however, be noted that it was previously reported ectopic recruitment of Tea4 to cell sides leads to Cdc42 activation and growth initiation from these sites (Kokkoris *et al*. 2014). As shown below, under this condition Tea4 is also colocalized with entrapped Tea1 (see below), which may account for these abnormal cell morphologies. Taken together, these results show that hyperphosphorylation of Tea1 through Cki3 prevents NETO onset and that conversely its hypophosphorylation promotes it.

### Tea4 dissociates from hyperphosphorylated Tea1

**Figure 4:**
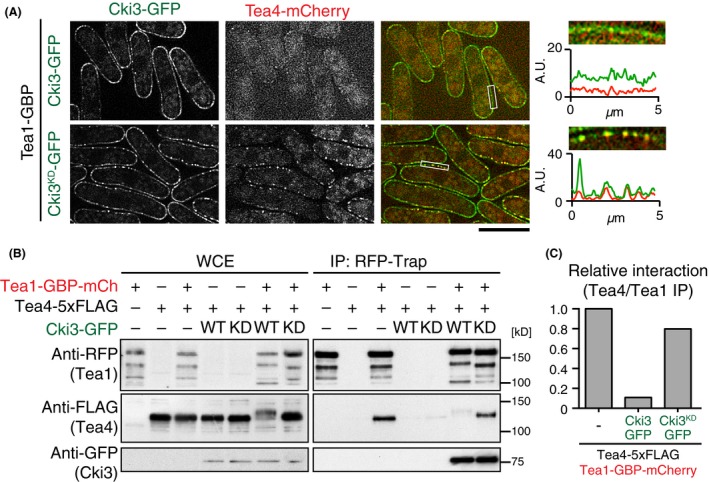
Tea1 entrapment by Cki3 results in physical dissociation of Tea4 from Tea1. (A) Representative images of Tea4‐mCherry and Cki3‐GFP or Cki3^KD^‐GFP in the presence of Tea1‐GBP. Density‐scan patterns of regions corresponding to white squares in the third panels from the left are shown on the far right corner: green (Cki3‐GFP/Cki3^KD^‐GFP) and red (Tea4‐mCherry). Scale bars, 10 μm. (B) Whole cell extracts were prepared from the indicated cells and pull‐down carried out with RFP‐Trap, followed by immunoblotting with anti‐FLAG, anti‐RFP, and anti‐GFP antibodies. (C) Quantification of immunoprecipitated Tea4 levels shown in (B). Tea1 levels are used as control.

Next, we investigated the underlying reason for Tea1 phosphorylation‐mediated unipolarity. It is known that Tea1 forms a stable complex with Tea4 and that in the absence of Tea1, Tea4 becomes delocalized from the cell tips (Martin *et al*. 2005; Tatebe *et al*. 2005). Given these preceding results, we examined Tea4 localization in cells containing Tea1‐GBP and Cki3‐GFP. Notably, Tea4 no longer colocalized with Tea1 at the plasma membrane, but instead it was dispersed throughout the cytoplasm (Fig. 4A, top). Interestingly, in contrast, in cells producing Tea1‐GBP and kinase‐negative Cki3^KD^‐GFP, Tea4 was localized to the plasma membrane together with Cki3^KD^ (Fig. 4A, bottom). Pull‐down experiments fully substantiated these data: Tea1‐GBP‐mCherry coprecipitated with only wild‐type Cki3‐GFP, but not Tea4, whereas it did interact with both Cki3^KD^‐GFP and Tea4 (Fig. 4B,C). These results suggested that monopolar growth in cells containing hyperphosphorylated Tea1 was ascribable to the dissociation of Tea4 from Tea1, recapitulating the deletion of Tea1. We also addressed whether Tea4 is dissociated from nongrowing tips during an unperturbed cell cycle. A wild‐type strain containing Tea1‐GFP and Tea4‐mCherry was grown on agar pad and time‐lapse live images were taken every 1 min in cells immediately after division that contain growing old ends and nongrowing new ends. Interestingly, quantification of Tea1 and Tea4 signals showed that these two proteins localized more abundantly to the old end than to the new end; however, Tea1 and Tea4 at the new end still appeared to colocalize as they did at the old end (Fig. S3 in Supporting Information). Thus, whether the dissociation of Tea4 from Tea1 takes place during an unperturbed cell cycle remains to be established. More work will be required to address this issue.

**Figure 5:**
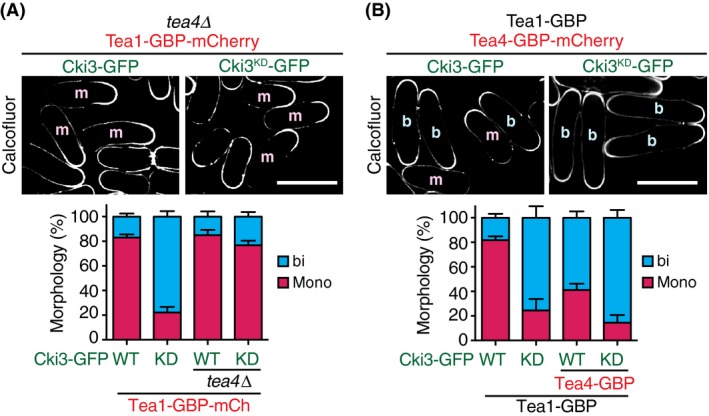
Tea4 is required for bipolar growth. (A) Tea4 is necessary for bipolar growth in a strain containing Cki3‐GFP ^KD^ and Tea1‐GBP‐mCherry. Growth polarity of each strain was determined with Calcofluor White staining. The percentage of monopolar (red) and bipolar (blue) cells were calculated and plotted (*n* > 200). (B) Tethering Tea4 in the background Cki3‐GFP and Tea1‐GBP‐mCherry renders cells bipolar. Indicated cells were grown and growth polarity was determined with Calcofluor White staining (*n* > 200).

If Tea4 dissociation was the reason for monopolar growth in Cki3‐Tea1 entrapment strains, the following two predictions should be fulfilled. The first proposition is that bipolar growth patterns in cells containing Tea1‐GBP and Cki3^KD^‐GFP would become monopolar when Tea4 is deleted. As shown in Fig. 5A, a strain containing *tea1‐GBPcki3* ^*KD*^ *‐GFPtea4*∆ exhibited mainly monopolar growth (80%). The second prediction is that the tethering of Tea4 to the plasma membrane in otherwise monopolar Tea1‐GBP and Cki3‐GFP cells would become bipolar. To address this, we created double GBP constructs, Tea1‐GBP and Tea4‐GBP in the Cki3‐GFP background. Interestingly, these cells exhibited NETO‐proficient, bipolar growth (Fig. 5B). Hence, localizing Tea4 to the plasma membrane was sufficient to convert growth patterns from monopolar to bipolar irrespective of the phosphorylation status of Tea1. Taking these results together, we propose that Tea1 phosphorylation through Cki3 at the cell tip plays an inhibitory role in NETO onset, which is attributable to compromised interaction with Tea4.

We then asked whether Tea4 could promote NETO using the similar entrapment system. However, no substantial changes in growth patterns in cells containing Tea4‐GBP‐mCherry in either a Cki3‐GFP or Cki3^KD^‐GFP background were observed (Fig. S4A,B in Supporting Information). Immunoblotting showed that Tea4 was hyperphosphorylated, which was dependent on Cki3 kinase activity (Fig. S4C in Supporting Information), and immunoprecipitation experiments indicated that in either a Cki3 or Cki3^KD^ background, Tea4 formed a complex with Cki3 and Tea1 (Fig. S4D in Supporting Information). This substantiates the notion that hyperphosphorylation of Tea1, but not that of Tea4, is responsible to regulate binding between these two proteins. Therefore, Tea4 is required but not sufficient to alter growth polarity in this entrapment system. Furthermore, *tea1* deletion in a strain containing Tea4‐GBP‐mCherry and Cki3‐GFP resulted in monopolar growth (Fig. S4E in Supporting Information), underscoring the important for Tea1. We surmise that Tea1 plays an additional key role in cell polarity control besides binding and recruitment of Tea4.

### Cki3‐mediated phosphorylation of Tea1 at five serine residues is critical for the timing of NETO onset

**Figure 6:**
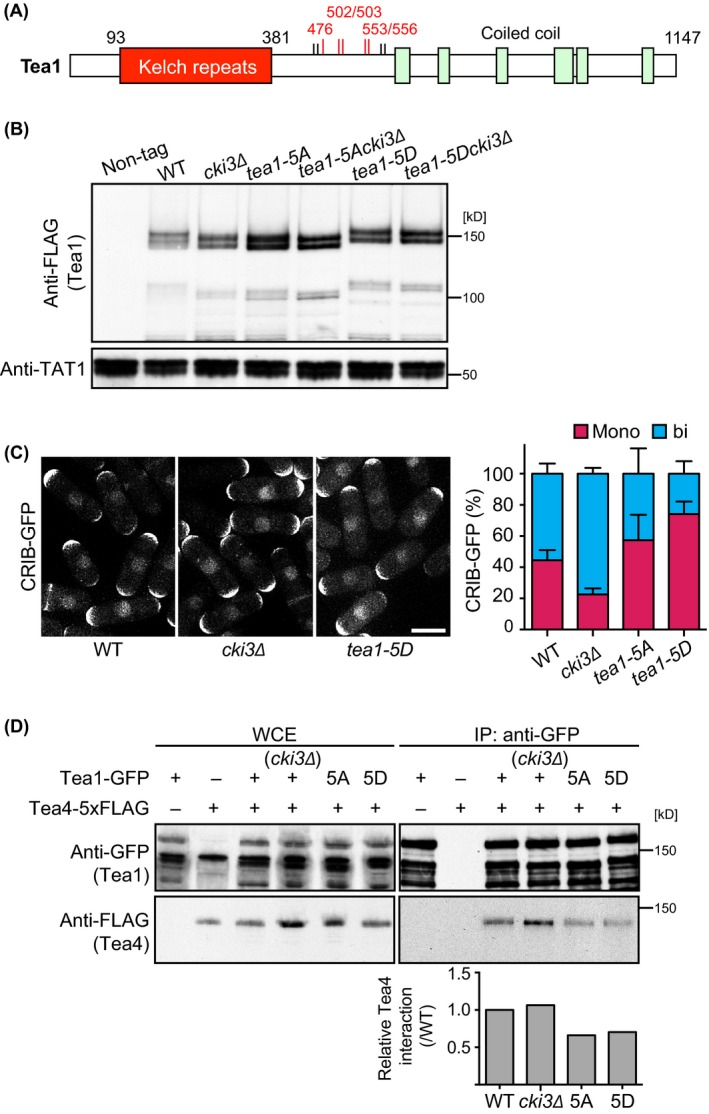
Phosphorylation of four serine residues in the middle region of Tea1 is Cki3 dependent and leads to monopolar growth. (A) Schematic presentation of Tea1 phosphorylation sites. Residues whose phosphorylation levels were significantly changed in *cki3*∆ cells are shown in red. (B) Whole cell extracts were prepared from each strain and immunoblotting carried out with anti‐FLAG and anti‐α‐tubulin antibodies. (C) Each of the indicated strains was grown to the exponential phase and growth polarity determined with CRIB‐GFP. Quantification data are shown at the right (*n* > 200). (D) Whole cell extracts were prepared from individual cells and immunoprecipitation carried out with anti‐GFP antibody, followed by immunoblotting with anti‐FLAG and anti‐GFP antibodies. Quantification of precipitated Tea4 is shown at the bottom.

To identity Cki3‐dependent phosphorylation sites within Tea1, we implemented semi‐quantitative liquid chromatography–mass spectrometry (LC–MS). Inspection of phosphopeptides identified nine phosphosites in WT, of which four sites (S502, S503, S553, S556) were hypophosphorylated in *cki3*∆ cells (Fig. 6A and Fig. S5A–C in Supporting Information). All these sites corresponded to the consensus phosphorylation sequence catalyzed by CK1, S/T/D/E x_1–3_ S/T (Knippschild *et al*. 2014; Fig. S6A in Supporting Information).

We then created Tea1 phosphomimetic and nonphosphorylatable mutants by replacing the four serines plus one additional canonical CK1 consensus site (S476) with aspartates and alanines, designated Tea1‐5D and Tea1‐5A, respectively (Fig. S6A in Supporting Information). Immunoblotting showed that Tea1‐5A ran faster in either wild‐type or *cki3*Δ cells and displayed a very similar pattern to that of Tea1 in *cki3*Δ cells (Fig. 6B). In contrast, the mobility of Tea1‐5D looked slower than that of Tea1 irrespective of wild‐type or *cki3*Δ background. Cells containing Tea1‐5D showed NETO delay (Fig. 6C) and compromised interaction with Tea4 (Fig. 6D and Fig. S6B in Supporting Information), characteristic of cells containing Cki3‐GFP and Tea1‐GBP‐mCherry (Fig. 3D). Tea1‐5D also effectively inhibited growth polarity transition of *cki3*Δ cells under S‐phase arrest condition (Fig. S6C in Supporting Information). These results are consistent with the idea that Tea1 phosphorylation through Cki3 is inhibitory toward NETO execution. On the contrary and rather unexpectedly, cells containing Tea1‐5A also displayed a similar monopolar growth instead of bipolar patterns under both unperturbed and S‐phase arrest conditions (Fig. 6C and Fig. S6B in Supporting Information). The reason for this discrepancy is currently not resolved (see Discussion). However, monopolar growth patterns conferred by Tea1‐5D highly suggested that Tea1 was negatively regulated by Cki3 for NETO execution in fission yeast.

## Discussion

In this work, we demonstrated that fission yeast CK1γ Cki3 played a decisive role in determining the timing of NETO onset, which was carried out through Tea1 phosphorylation. We found that Tea4 was dissociated from Tea1 upon Tea1 hyperphosphorylation and that tethering of Tea4 to the plasma membrane under this condition restored bipolar growth. We propose that Cki3 monitors the timing of NETO onset through Tea1 phosphorylation by regulating the interaction between Tea1 and Tea4. Five internal serine residues identified in this study were clustered around the central region. Previous work reported that the coiled coil‐rich C‐terminal region (538–1147) of Tea1 on its own is sufficient for the interaction with Tea4 *in vitro* (Martin *et al*. 2005). It is possible that the central region acts as a hinge, which may, when phosphorylated, mask the C‐terminal Tea4‐binding domain by the N‐terminal region through an intramolecular structural hindrance, which would be relieved by dephosphorylation.

**Figure 7:**
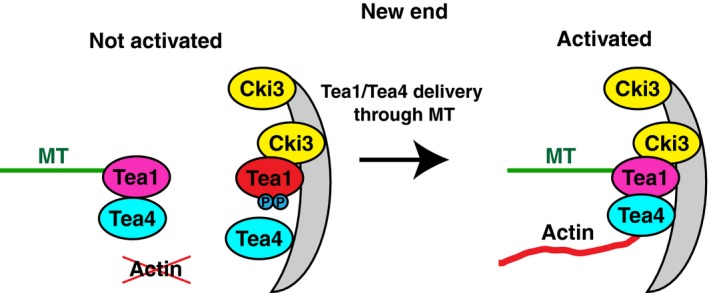
A speculative model. At the non‐growing new end (left), Tea1 is phosphorylated through membrane‐bound Cki3, thereby rendering it inactive for the potentiation of growth from this end. It is possible that under this condition, interaction between phospho‐Tea1 and Tea4 is compromised. When Tea1, which is dephosphorylated in a complex with Tea4, is delivered to this end through microtubules (right), this complex activates the actin‐mediated process, thereby triggering NETO. A proposal of the dissociation between Tea1 and Tea4 at the nongrowing ends under unperturbed conditions is hypothetical at the moment. For simplicity, not all polarity factors involved in NETO onset have been shown.

In contrast to the dissociation of Tea4 from hyperphosphorylated or phosphomimetic Tea1, under normal conditions, Tea1 and Tea4 are reported to constitutively colocalize to both growing and nongrowing ends (Martin *et al*. 2005; Tatebe *et al*. 2005). We confirmed this notion and furthermore found that Tea1 and Tea4 localized more strongly in the growing end than in the nongrowing end. However, the qualitative differences between these two ends in terms of localization of Tea1 and Tea4 were not discernable under our imaging conditions. We envision that Tea1 and Tea4 at nongrowing ends might dissociate within the cell tip microenvironments. Our results also put forward the proposition that the phosphorylation levels of Tea1 at old and new ends would be asymmetrically regulated in a cell cycle‐dependent manner. We envisage that Tea1 at the nongrowing new end would be phosphorylated through Cki3 and then when microtubules deliver polarity complexes containing additional Tea1–Tea4, in which Tea1 is dephosphorylated through Tea4‐PP1 (though at the moment this notion has not experimentally been shown), it now triggers new growth from this end by activating Cdc42 (depicted in Fig. 7).

In addition to during an unperturbed cell cycle, Cki3 also is required for NETO delay when DNA replication checkpoint is activated (Koyano *et al*. 2015). Under the latter condition, Cki3 acts downstream of Cds1 and calcineurin (Kume *et al*. 2011). However, as Cds1 is not involved in NETO regulation under a G1 arrest (Koyano *et al*. 2010) or during an unperturbed cell cycle (our unpublished observation), we envisage that the upstream regulators of Cki3 during the cell cycle would not be Cds1 or calcineurin. Identification of the upstream factors as well as proteins acting downstream of Tea1–Tea4 would be one of the future directions to be explored. A group of polarity factors that act downstream and/or regulate GTPases Cdc42 and Arf6 including Gef1^GEF^, Rga4^GAP^, Syt22^GEF^, Upc3^GAP^, and For3^Formin^ would be such effectors (Martin *et al*. 2007; Fujita 2008; Fujita & Misumi 2009, 2011; Huisman & Brunner 2011; Kokkoris *et al*. 2014; Das *et al*. 2015).

One conundrum with regard to Tea1 phosphorylation and NETO inhibition is that cells containing Tea1‐5A, which were expected to display bipolar growth, grew in a monopolar manner instead, indistinguishable from those containing Tea1‐5D or *tea1*Δ. This finding may be due to several reasons. First, the replacement of the five serines with alanines may have locally disrupted the Tea1 structure; coiled coil profiles predict that Tea1‐5A produces one abnormal coiled coil around this region (Fig. S6D in Supporting Information), suggesting that Tea1‐5A is not equivalent to nonphosphorylated Tea1. Second, perhaps a dynamic equilibrium and balance between phosphorylated and nonphosphorylated Tea1 are critical for NETO onset. The PP1‐binding property of Tea4 (Alvarez‐Tabares *et al*. 2007; Kokkoris *et al*. 2014) supports this notion. Third, there may be additional phosphorylation sites within Tea1 that are carried out by another kinase (Kim *et al*. 2003). Alternatively, substrate(s) other than Tea1 might be operational. It is also possible that the −A and −D substitutions result in the creation of loss‐of‐function Tea1 mutants independent of its phosphorylation status. These propositions are not necessarily mutually exclusive, and further analysis would be necessary to clarify this point.

In many eukaryotes, kelch‐repeat proteins and CK1γ are involved in cell polarity control and linked to several human diseases (Adams *et al*. 2000; Knippschild *et al*. 2014). In budding yeast, it is known that Kel1/Kel2 and Bud14, counterparts of Tea1 and Tea4, respectively, form a complex, thereby regulating cell polarization (Gould *et al*. 2014). CK1γ Yck1 and Yck2 are known to be required for cell morphogenesis (Robinson *et al*. 1999). In metazoans, functions of kelch‐repeat proteins are often linked to actin‐mediated cellular processes including cell polarization (Adams *et al*. 2000). In *C. elegans*, CK1γ regulates cell polarization through asymmetric spindle positioning (Panbianco *et al*. 2008), and in *Drosophila* and *Xenopus*, this kinase is required for proper embryogenesis by coupling Wnt receptor activation to cytoplasmic signal transduction (Davidson *et al*. 2005). However, except for fission yeast shown in this study, a functional relationship between CK1γ and the kelch‐repeat proteins remains to be investigated. It would be of great interest to explore this notion, and the results described in this work will be instrumental for forthcoming studies in other systems.

## Experimental procedures

### Yeast general methods

Standard media and methods for *S. pombe* were used (Moreno *et al*. 1991). Doubly tagged or mutant strains were constructed by tetrad dissection. Gene deletion and tagging were carried out with the PCR‐based method using homologous recombination at the corresponding genomic loci (Bähler *et al*. 1998; Sato *et al*. 2005). Fission yeast strains used in this study are listed in Table S1 in Supporting Information. Strains were grown in rich YE5S media and incubated at 27 °C unless otherwise stated.

### Microscopy and cell imaging

The DeltaVision RT system (Applied Precision) consisting of an Olympus IX70 wide‐field inverted fluorescence microscope, Olympus PlanApo ×100 (numerical aperture 1.4) and oil‐immersion objectives, and a CoolSNAP HQ camera (Roper Scientific), was used for observing protein localization and cell morphology. The images were captured and processed by iterative constrained deconvolution using SoftWoRx (Applied Precision). To observe cell morphology, 1 *μ*L of Calcofluor (5 mg/mL) was added in 500 *μ*L of cell cultures (final concentration: 1 *μ*g/mL) and briefly vortex, left at room temperature for 1 min. Cells were then collected by centrifuge (3000 rpm, 1 min) and mounted on 2% agar pad containing glutamate‐based minimal media (Moreno *et al*. 1991) with appropriate supplements. For live imaging of CRIB‐GFP, cells were mounted on 2% agar pad containing YE5S and left at 27 °C for 30 min, and then started imaging. The images were taken every 6 min in 10 Z‐sections of 0.4 *μ*m thicknesses each otherwise stated. The max projection images were used for data analysis. Cell length was measured by ImageJ software (National Institutes of Health, Bethesda, MD, USA).

### Immunochemistry

Preparation of cell extracts and immunoprecipitation were carried out as follows: 5 × 10^8^ cells were collected by centrifugation. All subsequent manipulations were carried out at 4 °C or on ice. Cells were broken in POM buffer (25 mm HEPES at pH 7.4, containing 0.1% Triton X‐100, 10% glycerol, 50 mm potassium acetate, 50 mm NaF, 60 mm β‐glycerolphosphate, 2 mm EDTA, 1 mm dithiothreitol, 0.1 mm sodium vanadate, 15 mm p‐nitrophenylphosphate, 40 *μ*g/mL aprotinin, 20 *μ*g/mL leupeptin, 1 *μ*g/mL pepstatin and 1 mm phenylmethylsulphonyl fluoride) with acid‐washed glass beads by FastPrep FP120 apparatus (5 × 25 s, power 5.5; BIO‐101, Inc., La Jolla, CA, USA). Extracts were cleared by centrifugation for 2 min at 7000 rpm. Protein concentrations were measured with a Bradford assay kit (Bio‐Rad, Hercules, CA, USA). For immunoprecipitation of Cki3‐HA (Fig. 2C), 6 mg of protein was pre‐treated with magnetizable beads conjugated to protein G (Dynabeads, DYNAL; Thermo Fisher Scientific, Waltham, MA, USA) by incubation at 4 °C with rotation for 60 min. The pre‐treated lysates were then separated from the beads and incubated with fresh protein G and a monoclonal anti‐HA antibody (16B12; BAbCO, Berkeley, CA, USA) at 4 °C for 2 h. For pull‐down of GFP‐ or GBP‐mCherry‐tagged strains, 6 mg of proteins was incubated with GFP‐Trap or RFP‐Trap, respectively, (ChromoTek GmbH, Martinsried, Germany) at 4 °C for 90 min with rotation. The beads were then washed in POM buffer, and cell extracts or immunocomplexes bound to them were separated on SDS‐PAGE and analyzed by immunoblotting. The λ‐phosphatase (λ‐PPase; New England Biolabs, Inc., Beverly, MA, USA) was used for phosphatase treatment. Cell extracts were incubated at 30 °C for 1 h in the presence or absence of λ‐PPase (400 units). Antibody probes used for immunoblotting were as follows: anti‐HA (16B12; BAbCO), anti‐GFP (Roche Holding AG, Basel, Switzerland), anti‐GFP (rabbit polyclonal, AMS Biotechnology, UK), anti‐RFP (rabbit polyclonal, 600‐401‐379; Rockland Immunochemicals, Gilbertsville, PA, USA), anti‐FLAG (M2, Sigma‐Aldrich, St. Louis, MO, USA), and anti‐α‐tubulin (TAT‐1, provided by K. Gull, Oxford University, UK) antibodies.

### Identification of phosphorylation sites within Tea1 by LC‐MS

To identity Cki3‐dependent phosphorylation sites within Tea1, we implemented semi‐quantitative liquid chromatography–mass spectrometry (LC–MS). Endogenous Tea1 was immunoprecipitated from wild‐type or *cki3*∆ cells. Corresponding bands were cut out from SDS‐PAGE gels upon colloidal Coomassie staining, digested with trypsin and analyzed by LC–MS. Inspection of phosphopeptides between these two preparations using the Matrix Science Mascot and Andromeda MaxQuant (Cox *et al*. 2011) search engines and Skyline software (Schilling *et al*. 2012) identified nine phosphosites in WT samples, of which four sites (S502, S503, S553, S556) were hypophosphorylated in *cki3*∆ cells (Fig. S5A–D in Supporting Information). All these sites corresponded to the consensus phosphorylation sequence catalyzed by CK1, S/T/D/E x_1–3_ S/T, in which the first S/T is phosphorylated by another priming kinase. It is noteworthy that all phosphorylation sites are clustered around the central region that is situated between the *N*‐terminal kelch‐repeat and the *C*‐terminal domain rich in coiled coils (Fig. 6A).

### Statistical data analysis

All *P*‐values are from two‐tailed unpaired Student's *t*‐tests. Unless otherwise stated, we followed this key for asterisk placeholders for *P*‐values in the figures: ****P < 0.0001.

## Supporting information


**Figure S1** *cki3Δ* cells undergo premature NETO.
**Figure S2** Tea1 is hypophosphorylated in *cki3Δ* cells.
**Figure S3** Localization of Tea1 and Tea4 at the old and new ends.
**Figure S4** Tea4 is incapable of altering growth patterns when tethered to the plasma membrane through Cki3.
**Figure S5** Identification of Cki3‐dependent phosphorylated residues within Tea1.
**Figure S6** Analysis of phosphomimetic and nonphosphorylatable Tea1 mutants.
**Table S1** Strain list in this study

## References

[gtc12309-bib-0001] Adams, J. , Kelso, R. & Cooley, L. (2000) The kelch repeat superfamily of proteins: propellers of cell function. Trends Cell Biol. 10, 17–24. 10603472 10.1016/s0962-8924(99)01673-6

[gtc12309-bib-0002] Alvarez‐Tabares, I. , Grallert, A. , Ortiz, J.M. & Hagan, I.M. (2007) *Schizosaccharomyces pombe* protein phosphatase 1 in mitosis, endocytosis and a partnership with Wsh3/Tea4 to control polarised growth. J. Cell Sci. 120, 3589–3601. 17895368 10.1242/jcs.007567

[gtc12309-bib-0003] Bähler, J. , Wu, J. , Longtine, M.S. , Shah, N.G. , McKenzie, A. III , Steever, A.B. , Wach, A. , Philippsen, P. & Pringle, J.R. (1998) Heterologous modules for efficient and versatile PCR‐based gene targeting in *Schizosaccharomyces pombe* . Yeast 14, 943–951. 9717240 10.1002/(SICI)1097-0061(199807)14:10<943::AID-YEA292>3.0.CO;2-Y

[gtc12309-bib-0004] Chang, F. & Martin, S.G. (2009) Shaping fission yeast with microtubules. Cold Spring Harb. Perspect. Biol. 1, a001347. 20066076 10.1101/cshperspect.a001347 PMC2742080

[gtc12309-bib-0005] Cox, J. , Neuhauser, N. , Michalski, A. , Scheltema, R.A. , Olsen, J.V. & Mann, M. (2011) Andromeda: a peptide search engine integrated into the MaxQuant environment. J. Proteome Res. 10, 1794–1805. 21254760 10.1021/pr101065j

[gtc12309-bib-0006] Das, M. , Nunez, I. , Rodriguez, M. , Wiley, D.J. , Rodriguez, J. , Sarkeshik, A. , Yates, J.R. III , Buchwald, P. & Verde, F. (2015) Phosphorylation‐dependent inhibition of Cdc42 GEF Gef1 by 14‐3‐3 protein Rad24 spatially regulates Cdc42 GTPase activity and oscillatory dynamics during cell morphogenesis. Mol. Biol. Cell. 26, 3520–3534. 26246599 10.1091/mbc.E15-02-0095 PMC4591695

[gtc12309-bib-0007] Davidson, G. , Wu, W. , Shen, J. , Bilic, J. , Fenger, U. , Stannek, P. , Glinka, A. & Niehrs, C. (2005) Casein kinase 1 γ couples Wnt receptor activation to cytoplasmic signal transduction. Nature 438, 867–872. 16341016 10.1038/nature04170

[gtc12309-bib-0008] Estravis, M. , Rincon, S. & Perez, P. (2012) Cdc42 regulation of polarized traffic in fission yeast. Commun. Integr. Biol. 5, 370–373. 23060961 10.4161/cib.19977 PMC3460842

[gtc12309-bib-0009] Fujita, A. (2008) ADP‐ribosylation factor arf6p may function as a molecular switch of new end take off in fission yeast. Biochem. Biophys. Res. Commun. 366, 193–198. 18060866 10.1016/j.bbrc.2007.11.117

[gtc12309-bib-0010] Fujita, A. & Misumi, Y. (2009) Fission yeast syt22 protein, a putative Arf guanine nucleotide exchange factor, is necessary for new end take off. FEMS Microbiol. Lett. 294, 191–197. 19431238 10.1111/j.1574-6968.2009.01566.x

[gtc12309-bib-0011] Fujita, A. & Misumi, Y. (2011) Fission yeast *ucp3* gene encodes a putative Arf6 GTPase‐activating protein. Mol. Biol. Rep. 38, 3875–3882. 21107719 10.1007/s11033-010-0503-6

[gtc12309-bib-0012] Gould, C.J. , Chesarone‐Cataldo, M. , Alioto, S.L. , Salin, B. , Sagot, I. & Goode, B.L. (2014) *Saccharomyces cerevisiae* Kelch proteins and Bud14 protein form a stable 520‐kDa formin regulatory complex that controls actin cable assembly and cell morphogenesis. J. Biol. Chem. 289, 18290–18301. 24828508 10.1074/jbc.M114.548719 PMC4140247

[gtc12309-bib-0013] Huisman, S. & Brunner, D. (2011) Cell polarity in fission yeast: a matter of confining, positioning, and switching growth zones. Semin. Cell Dev. Biol. 22, 799–805. 21803169 10.1016/j.semcdb.2011.07.013

[gtc12309-bib-0014] Kim, H. , Yang, P. , Catanuto, P. , Verde, F. , Lai, H. , Du, H. , Chang, F. & Marcus, S. (2003) The kelch repeat protein, Tea1, is a potential substrate target of the p21‐activated kinase, Shk1, in the fission yeast, *Schizosaccharomyces pombe* . J. Biol. Chem. 278, 30074–30082. 12764130 10.1074/jbc.M302609200

[gtc12309-bib-0015] Knippschild, U. , Kruger, M. , Richter, J. , Xu, P. , Garcia‐Reyes, B. , Peifer, C. , Halekotte, J. , Bakulev, V. & Bischof, J. (2014) The CK1 family: contribution to cellular stress response and its role in carcinogenesis. Front. Oncol. 4, 96. 24904820 10.3389/fonc.2014.00096 PMC4032983

[gtc12309-bib-0016] Kokkoris, K. , Castro, D.G. & Martin, S.G. (2014) Tea4‐PP1 landmark promotes local growth by dual Cdc42 GEF recruitment and GAP exclusion. J. Cell Sci. 127, 2005–2016. 24554432 10.1242/jcs.142174

[gtc12309-bib-0017] Koyano, T. , Konishi, M. , Martin, S.G. , Ohya, Y. , Hirata, D. , Toda, T. & Kume, K. (2015) Casein kinase 1γ ensures monopolar growth polarity under incomplete DNA replication downstream of Cds1 and calcineurin in fission yeast. Mol. Cell. Biol. 35, 1533–1542. 25691662 10.1128/MCB.01465-14 PMC4387218

[gtc12309-bib-0018] Koyano, T. , Kume, K. , Konishi, M. , Toda, T. & Hirata, D. (2010) Search for kinases related to transition of growth polarity in fission yeast. Biosci. Biotechnol. Biochem. 74, 1129–1133. 20501954 10.1271/bbb.100223

[gtc12309-bib-0019] Kume, K. , Koyano, T. , Kanai, M. , Toda, T. & Hirata, D. (2011) Calcineurin ensures a link between the DNA replication checkpoint and microtubule‐dependent polarized growth. Nat. Cell Biol. 13, 234–242. 21336311 10.1038/ncb2166

[gtc12309-bib-0020] Martin, S.G. , McDonald, W.H. , Yates, J.R. & Chang, F. (2005) Tea4p links microtubule plus ends with the formin For3p in the establishment of cell polarity. Dev. Cell 8, 479–491. 15809031 10.1016/j.devcel.2005.02.008

[gtc12309-bib-0021] Martin, S.G. , Rincon, S.A. , Basu, R. , Perez, P. & Chang, F. (2007) Regulation of the formin for3p by cdc42p and bud6p. Mol. Biol. Cell 18, 4155–4167. 17699595 10.1091/mbc.E07-02-0094 PMC1995706

[gtc12309-bib-0022] Mata, J. & Nurse, P. (1997) Tea1 and the microtubular cytoskeleton are important for generating global spatial order within the fission yeast cell. Cell 89, 939–949. 9200612 10.1016/s0092-8674(00)80279-2

[gtc12309-bib-0023] McCaffrey, L.M. & Macara, I.G. (2009) Widely conserved signaling pathways in the establishment of cell polarity. Cold Spring Harb. Perspect. Biol. 1, a001370. 20066082 10.1101/cshperspect.a001370 PMC2742088

[gtc12309-bib-0024] Mitchison, J.M. & Nurse, P. (1985) Growth in cell length in the fission yeast *Schizosaccharomyces pombe* . J. Cell Sci. 75, 357–376. 4044680 10.1242/jcs.75.1.357

[gtc12309-bib-0025] Moreno, S. , Klar, A. & Nurse, P. (1991) Molecular genetic analysis of fission yeast *Schizosaccharomyces pombe* . Methods Enzymol. 194, 795–823. 2005825 10.1016/0076-6879(91)94059-l

[gtc12309-bib-0026] Panbianco, C. , Weinkove, D. , Zanin, E. , Jones, D. , Divecha, N. , Gotta, M. & Ahringer, J. (2008) A casein kinase 1 and PAR proteins regulate asymmetry of a PIP_2_ synthesis enzyme for asymmetric spindle positioning. Dev. Cell 15, 198–208. 18694560 10.1016/j.devcel.2008.06.002 PMC2686839

[gtc12309-bib-0027] Robinson, L.C. , Bradley, C. , Bryan, J.D. , Jerome, A. , Kweon, Y. & Panek, H.R. (1999) The Yck2 yeast casein kinase 1 isoform shows cell cycle‐specific localization to sites of polarized growth and is required for proper septin organization. Mol. Biol. Cell 10, 1077–1092. 10198058 10.1091/mbc.10.4.1077 PMC25234

[gtc12309-bib-0028] Rothbauer, U. , Zolghadr, K. , Muyldermans, S. , Schepers, A. , Cardoso, M.C. & Leonhardt, H. (2008) A versatile nanotrap for biochemical and functional studies with fluorescent fusion proteins. Mol. Cell Proteomics 7, 282–289. 17951627 10.1074/mcp.M700342-MCP200

[gtc12309-bib-0029] Sato, M. , Dhut, S. & Toda, T. (2005) New drug‐resistant cassettes for gene disruption and epitope tagging in *Schizosaccharomyces pombe* . Yeast 22, 583–591. 15942936 10.1002/yea.1233

[gtc12309-bib-0030] Schilling, B. , Rardin, M.J. , MacLean, B.X. , Zawadzka, A.M. , Frewen, B.E. , Cusack, M.P. , Sorensen, D.J. , Bereman, M.S. , Jing, E. , Wu, C.C. , Verdin, E. , Kahn, C.R. , Maccoss, M. J. & Gibson, B.W. (2012) Platform‐independent and label‐free quantitation of proteomic data using MS1 extracted ion chromatograms in skyline: application to protein acetylation and phosphorylation. Mol. Cell Proteomics 11, 202–214. 22454539 10.1074/mcp.M112.017707 PMC3418851

[gtc12309-bib-0031] Snaith, H.A. & Sawin, K.E. (2003) Fission yeast mod5p regulates polarized growth through anchoring of tea1p at cell tips. Nature 423, 647–651. 12789340 10.1038/nature01672

[gtc12309-bib-0032] Snaith, H.A. , Thompson, J. , Yates, J.R. III & Sawin, K.E. (2011) Characterization of Mug33 reveals complementary roles for actin cable‐dependent transport and exocyst regulators in fission yeast exocytosis. J. Cell Sci. 124, 2187–2199. 21652630 10.1242/jcs.084038 PMC3113670

[gtc12309-bib-0033] Tatebe, H. , Nakano, K. , Maximo, R. & Shiozaki, K. (2008) Pom1 DYRK regulates localization of the Rga4 GAP to ensure bipolar activation of Cdc42 in fission yeast. Curr. Biol. 18, 322–330. 18328707 10.1016/j.cub.2008.02.005 PMC2277499

[gtc12309-bib-0034] Tatebe, H. , Shimada, K. , Uzawa, S. , Morigasaki, S. & Shiozaki, K. (2005) Wsh3/Tea4 is a novel cell‐end factor essential for bipolar distribution of Tea1 and protects cell polarity under environmental stress in *S. pombe* . Curr. Biol. 15, 1006–1015. 15936270 10.1016/j.cub.2005.04.061

