# Supplementary material for: Casein kinase 1γ acts as a molecular switch for cell polarization through phosphorylation of the polarity factor Tea1 in fission yeast
Source: Genes Cells. 2015 Nov 2;20(12):1046–58. doi: 10.1111/gtc.12309 (PMC4737401; doi:10.1111/gtc.12309)
Supplement: Supplementary file 1 — Figure S1 cki3Δ cells undergo premature NETO. Figure S2 Tea1 is hypophosphorylated in cki3Δ cells. Figure S3 Localization of Tea1 and Tea4 at the old and new ends. Figure S4 Tea4 is incapable of altering growth patterns when tethered to the plasma membrane through Cki3. Figure S5 Identification of Cki3‐dependent phosphorylated residues within Tea1. Figure S6 Analysis of phosphomimetic and nonphosphorylatable Tea1 mutants. Table S1 Strain list in this study [file GTC-20-1046-s001.pdf]

Supporting Information

Casein kinase 1γ acts as a molecular switch for cell polarization through phosphorylation of the polarity factor Tea1 in fission yeast

Takayuki Koyano, Karin Barnouin, Ambrosius P. Snijders, Kazunori Kume, Dai Hirata and Takashi Toda

Supplemental Figures

Figure S1: *cki3Δ* cells undergo premature NETO

Figure S2: Tea1 is hypo-phosphorylated in *cki3Δ* cells

Figure S3 Localization of Tea1 and Tea4 at the old and new ends

Figure S4 Tea4 is incapable of altering growth patterns when tethered to the plasma membrane through Cki3

Figure S5: Identification of Cki3-dependent phosphorylated residues within Tea1

Figure S6: Analysis of phospho-mimetic and non-phosphorylatable Tea1 mutants

Supplemental Table S1

Supplemental References

Supplemental Figure S1

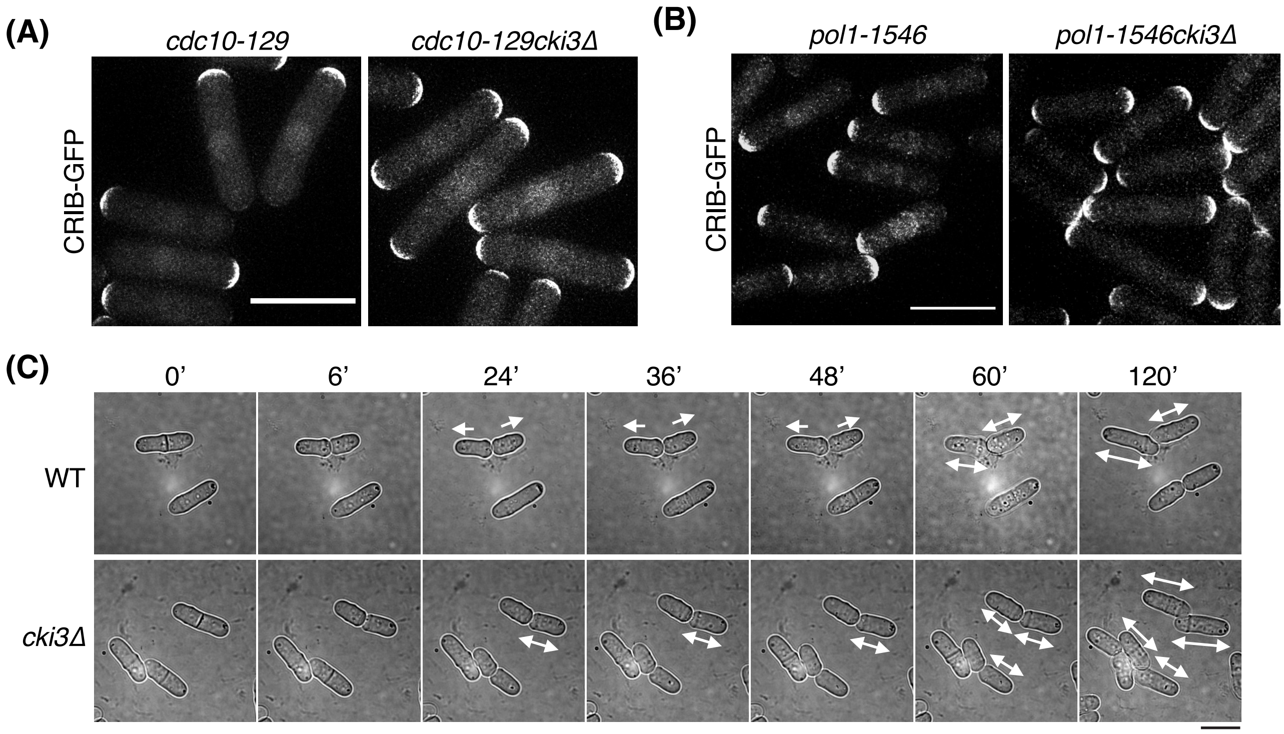

(D)

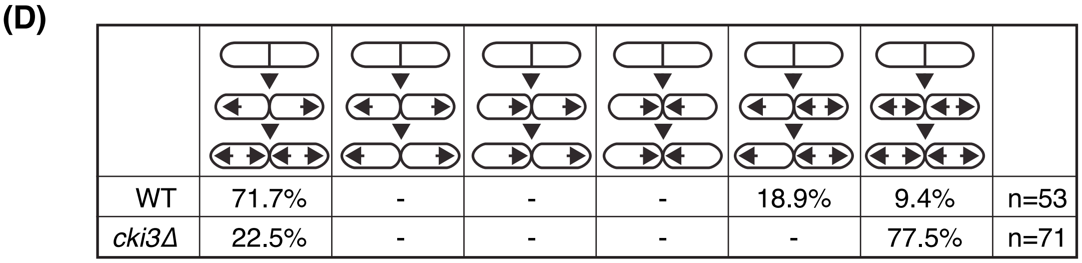

WT	71.7%	-	-	-	18.9%	9.4%	n=53
cki3Δ	22.5%	-	-	-	-	77.5%	n=71

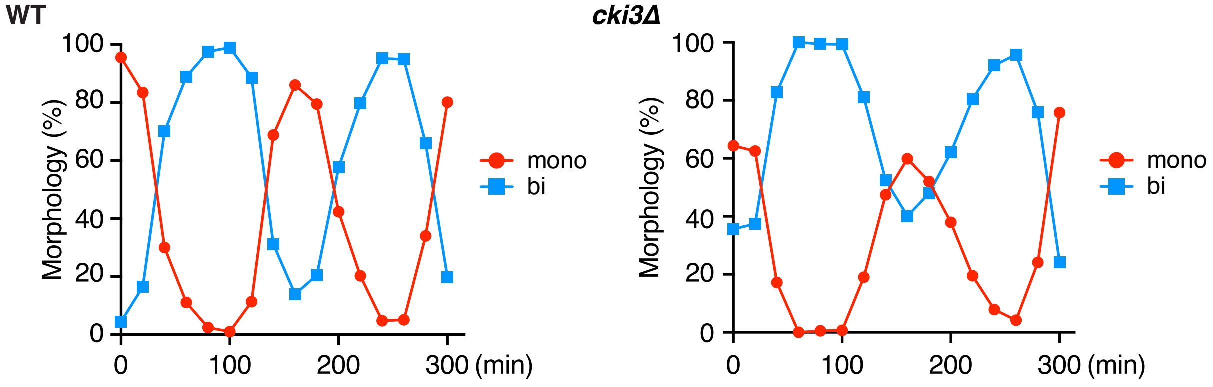

Supplemental Figure S1

***cki3Δ* cells undergo premature NETO**

(A, B) Exponentially growing *cdc10-129* (A) or *pol1-1546* mutant cells (B) containing CRIB-GFP in the presence (left) or absence (right) of the *cki3*$^+$ gene were shifted from 27ºC to 36ºC and incubated for an additional 4 h. In the single *cdc10* or *pol1* mutant, cells grew only from one end, to which CRIB-GFP was localized. In sharp contrast, *cdc10cki3Δ* or *pol1cki3Δ* double mutant cells displayed bipolar growth patterns. (C) Time-lapse images of growing wild type and *cki3Δ* cells are shown. Cells were grown on YE5S agar pad at 27ºC, and pictures were taken every six min. White arrows indicate the direction of cell growth. In wild type cells (top), NETO initiated at 60 min, while in *cki3Δ* cells at 24 min time-point, NETO started. (D) The percentage of cells with individual growth patterns in wild type and in *cki3Δ* mutants. This data is derived from time-lapse images shown in (C). (E) Centrifugal elutriation was performed on exponentially growing wild type (top) or *cki3Δ* (bottom) cells, by which small early G2 cells were collected. These cells were then incubation at 28ºC for the indicated times. At each time-point, aliquots of cells were taken and stained with Calcofluor White to monitor growth patterns. The percentage of monopolar (red) and bipolar (blue) cells were plotted. Note that at time 0, ~100% of the wild type cells displayed monopolar growth, whereas ~40% of *cki3Δ* cells had already committed NETO (bipolar growth).

Supplemental Figure S2

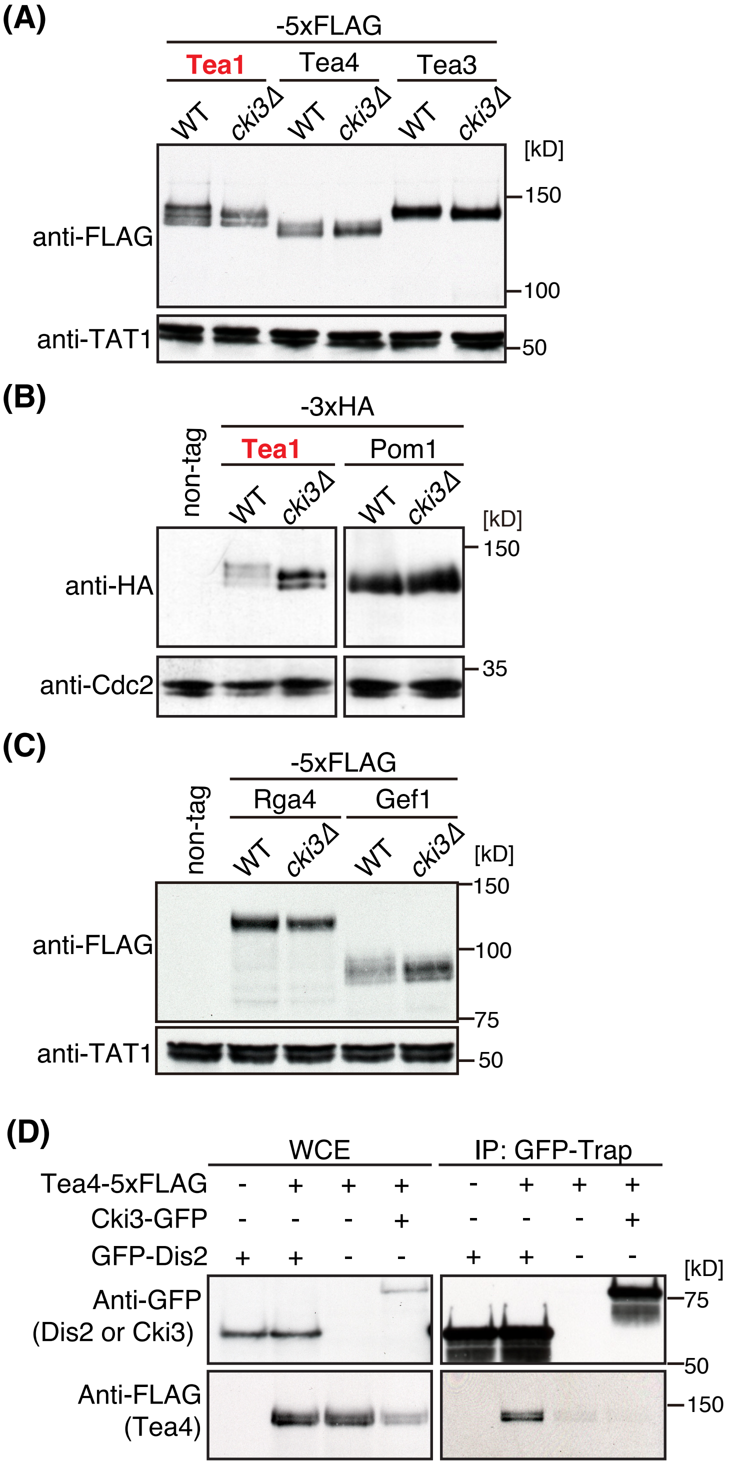

Supplemental Figure S2
Tea1 is hypo-phosphorylated in *cki3Δ* cells
(A-C) Six known polarity factors (Tea1, Tea3, Tea4, Pom1, Rga4 and Gef1) (Mata and Nurse, 1997; Bähler and Pringle, 1998; Arellano et al., 2002; Coll et al., 2003; Niccoli et al., 2003; Das et al., 2007; Das et al., 2012) known to be involved in NETO regulation were tagged with 5xFLAG or 3xHA at their C-termini and produced under each endogenous promoter in wild type and *cki3Δ* cells. Protein extracts were prepared from each strain and run on SDS-PAGE, and immunoblotting performed with anti-FLAG or anti-HA antibodies. The positions of molecular weight markers are indicated on the right. Only the Tea1 protein showed a clear difference in mobility; it ran faster in *cki3Δ* cells than in wild type ones. The appearance of two closely moving bands of Tea1 were reported previously, which were attributed to the presence of two closely situated ATGs at the N-terminal region (Mata and Nurse, 1997), and therefore these bands do not stem from the differences in the phosphorylation state.
(D) Tea4 did not interact with Cki3. Pull-down experiments were performed by using cell extracts prepared from the indicated strains. GFP-trap was applied, followed by immunoblotting with anti-GFP and anti-FLAG antibodies. Note that Tea4 interacted with PP1^{Dis2}, as previously reported (Alvarez-Tabares et al., 2007), but not with Cki3.

Supplemental Figure S3

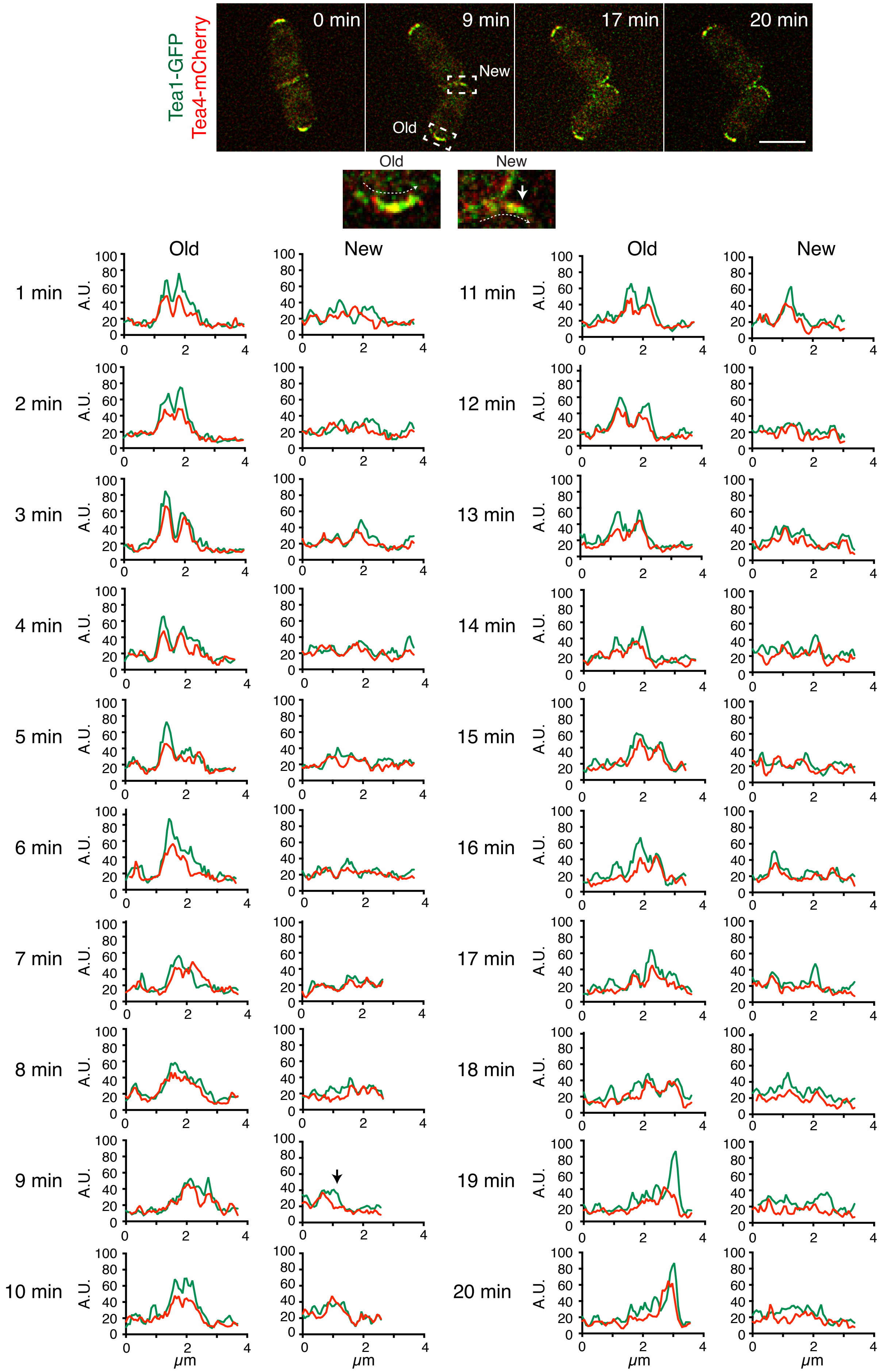

Supplemental Figure S3

Localization of Tea1 and Tea4 at the old and new ends

Cells containing Tea1-GFP and Tea4-mCherry were grown on GMM containing agar pad at 27ºC and time-lapse images were taken every 1 min. Five Z-sections of 0.3 μm thicknesses in the middle plane of each cell were recorded. Signal intensities of these two proteins were taken and signals intensities were quantified by using Image J. Representative images are shown (top and enlarged images of the regions corresponding to new and old ends are shown below). Quantification of signal intensities of Tea1 (green) and Tea4 (red) at the new and old ends during 20 min imaging are shown.

Supplemental Figure S4

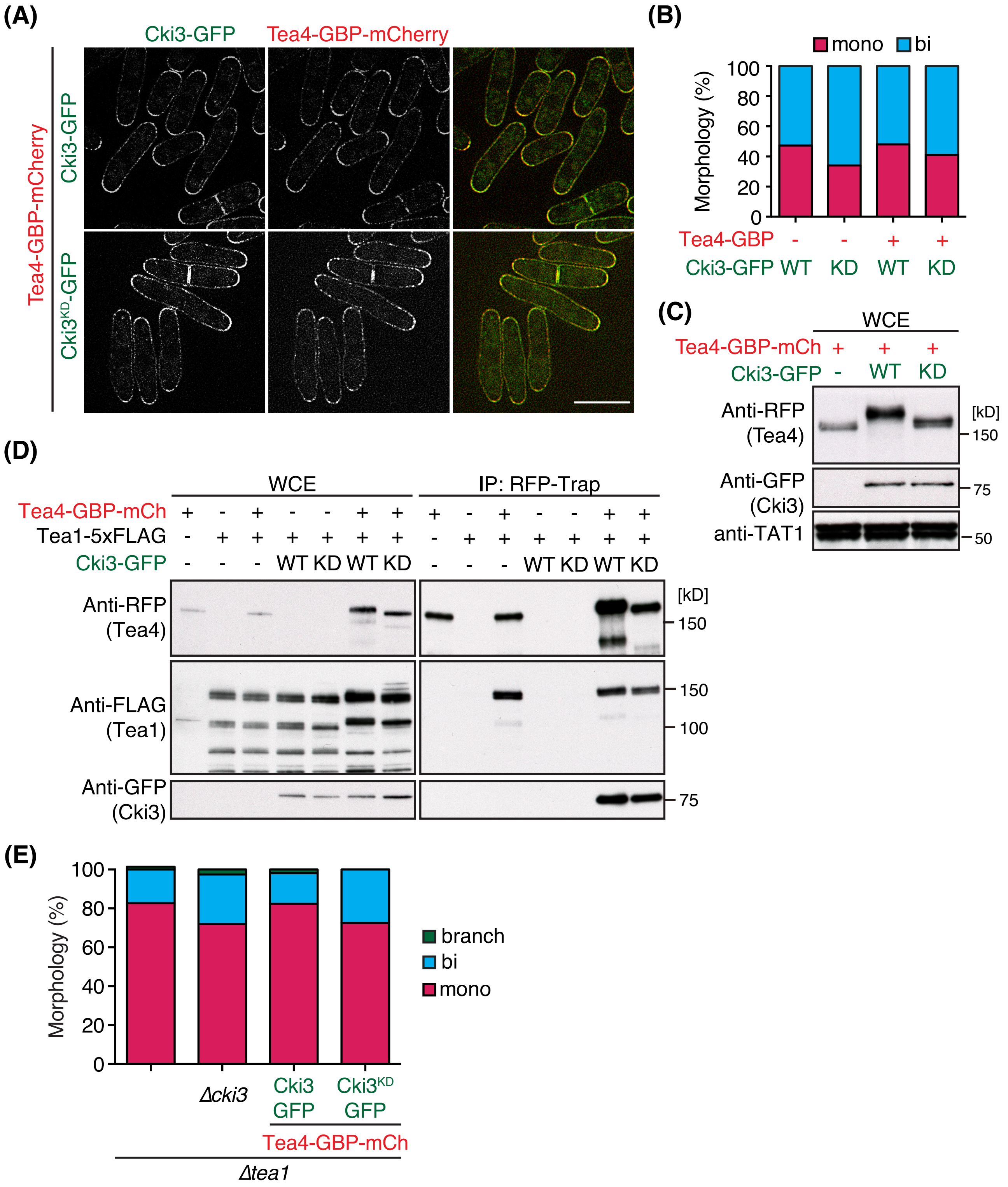

Supplemental Figure S4

Tea4 is incapable of altering growth patterns when tethered to the plasma membrane through Cki3

(A) Cells containing Tea4-GBP-mCherry and Cki3-GFP (top) or Cki3KD-GFP (bottom) were grown exponentially and observed under a fluorescence microscope. Scale bar, 10 μm. (B) Tethering Tea4 is not sufficient for bipolar growth. Growth polarity in the indicated strains was examined with Calcofluor White staining (n>200). (C) Immunoblotting was performed by using the cell extracts shown in (A). Antibodies against RFP, GFP and α-tubulin were applied. Tea4 is hyper-phosphorylated in cells containing Tea4-GBP-mCherry and Cki3-GFP. (D) Pull-down experiments were performed by using RFP-trap and cell extracts prepared from the indicated cells containing various combinations of tagged constructs. Pulled-down precipitates were immunoblotted with antibodies against RFP (Tea4), FLAG (Tea1) and GFP (Cki3). (E) Tea1 is required for bipolar growth of strains in which Tea4 is entrapped by Cki3 (Cki3-GFP Tea4-GBP-mCherry). Growth polarity in the indicated strains was examined with Calcofluor White staining (n>200).

Supplemental Figure S5

(A)

PEP	Modified sequence	Localization probability	Positions in Protein	Score	Number of Phospho (STY)
0.008013	_HQT(ph)ISTPVSGR_	0.998537	452	57	1
1.74E-05	_ASNDLPS(ph)PVVPTR_	0.999915	467	108	1
7.71E-13	_SNS(ph)SSILQPSYNLNSHSSDRR_	0.835201	476	115	1
2.41E-17	_NTNDDDQS(ph)SLNSQQLSNQAK_	0.49992	502	134	1
2.41E-17	_NTNDDDQSS(ph)LNSQQLSNQAK_	0.71747	503	134	1
8.05E-05	_S(ph)INSISEVSEVR_	0.999616	553	105	1
1.84E-10	_SINS(ph)ISEVSEVRFPEQSSVK_	0.995649	556	103	1
0.013144	_TVDERKS(ph)LDGR_	0.996828	579	57	1
8.87E-10	_ITS(ph)VTLETLVEK_	0.999255	586	145	1

(B)

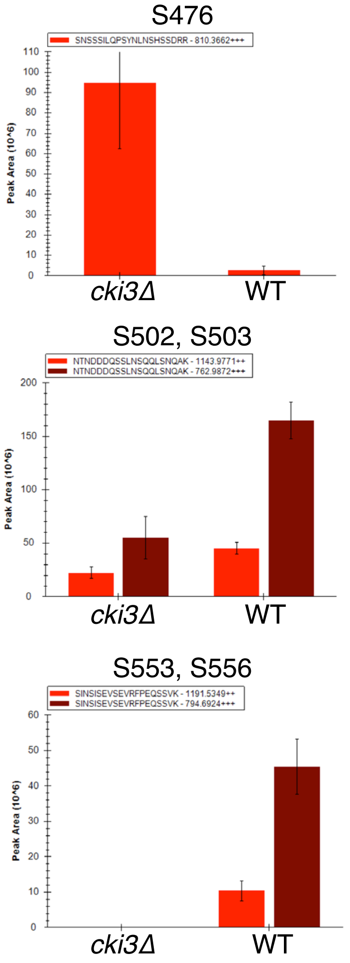

(C)

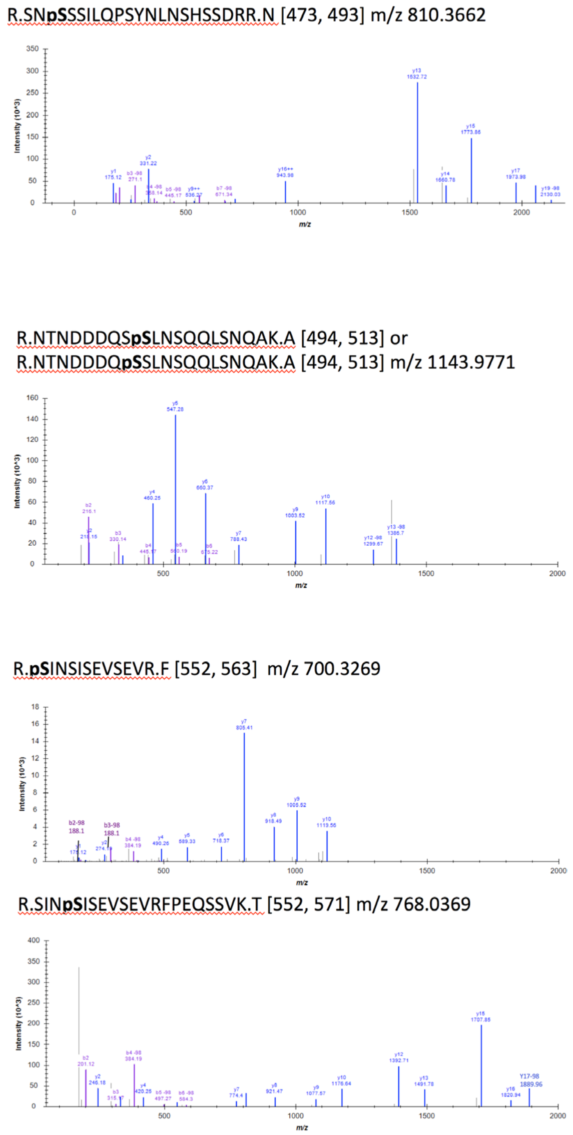

Supplemental Figure S5

Identification of Cki3-dependent phosphorylated residues within Tea1

(A) Table of identified phosphopeptides. Phosphorylated serines and results of statistical evaluation are shown. Four serine residues out of nine serines were hypo-phosphorylated in samples prepared from *cki3Δ* cells. In red are the phosphorylation sites that have been identified to produce a phenotype following site-directed mutagenesis. (B) Semi-quantitative analysis of peptides containing phosphorylated serines performed in Skyline. All samples were analysed in triplicate by LC-MS. Shown are the mean peak areas +/- SD. Numbers are the phosphorylation sites identified to produce a phenotype following site-directed mutagenesis. (C) MS/MS sequence spectra of phosphorylated Tea1 peptides. -98 denotes loss of H_3PO_4 and confirms the presence of phosphate. The phosphorylation sites are those identified to produce a phenotype following site-directed mutagenesis. The phosphorylated (p) sites are highlighted in bold type within the peptide sequence.

Supplemental Figure S6

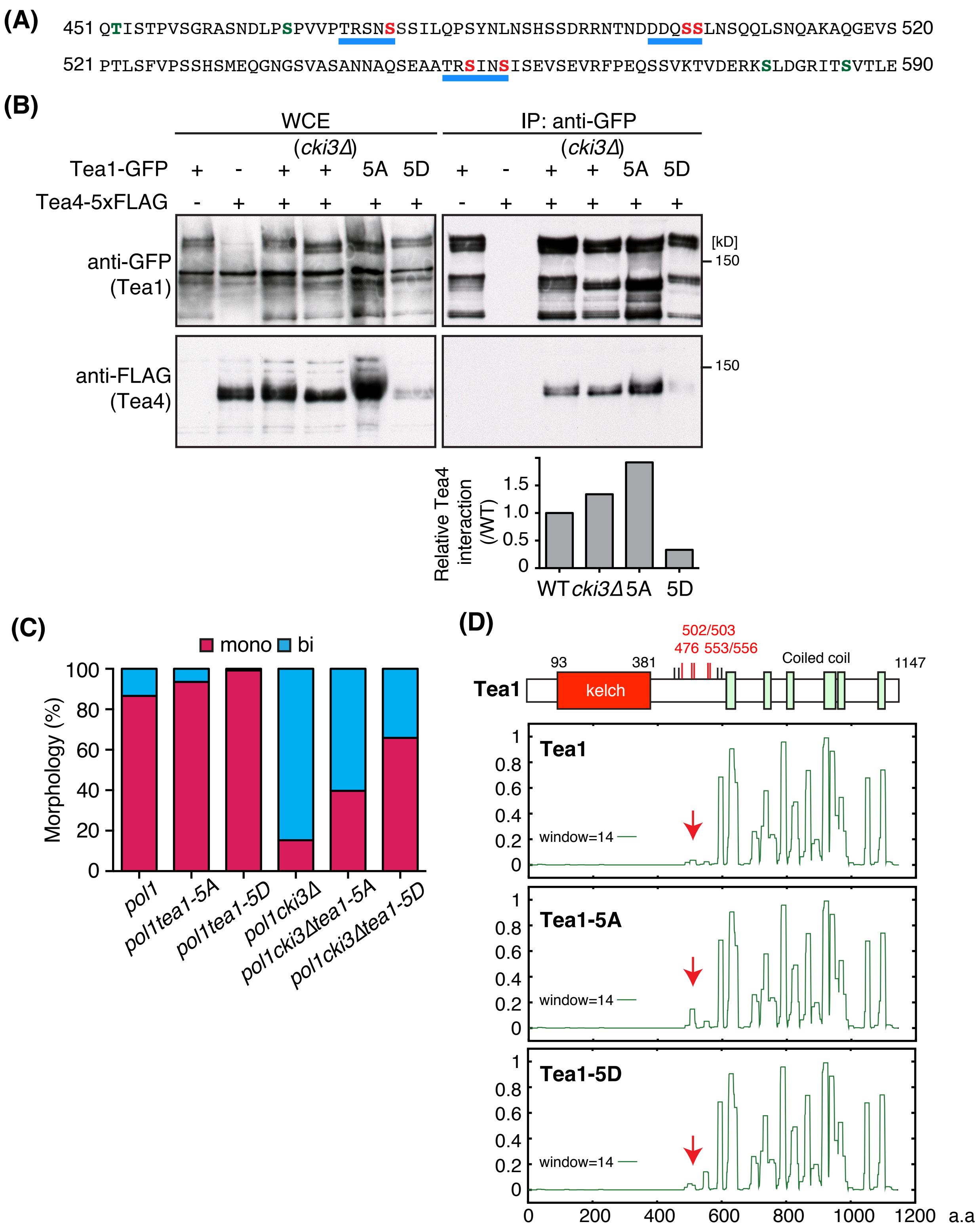

Supplemental Figure S6

Analysis of phospho-mimetic and non-phosphorylatable Tea1 mutants

(A) Amino acid sequence encompassing phosphorylation sites within Tea1. Five serines (shown in red), which were mutated, corresponded to the consensus phosphorylation sequence catalyzed by CK1, S/T/D/E x1-3 S/T, in which the first S/T is phosphorylated by another priming (Knippschild et al., 2005; Knippschild et al., 2014). (B) Whole cell extracts were prepared from the individual strains and immunoprecipitation performed with anti-GFP antibody, followed by immunoblotting with anti-FLAG and anti-GFP antibodies. Quantification of precipitated Tea4 is shown at the bottom. This is an independent duplicate experiment shown in Fig. 6D. (C) The indicated mutant cells containing CRIB-GFP were cultured at 27ºC and shifted to 36ºC and incubated for an additional 4 h. *pol1cki3Δ* double mutant cells displayed bipolar growth patterns, however those of *pol1cki3Δtea1-5D* triple mutant increased monopolar cells (n>150). (D) An overall structure of Tea1 is shown on the top with assigned phosphorylation sites. Positions of phosphorylation sites identified by LC-MS are indicated with vertical lines, in which five residues that were mutated are shown with red lines (the same as Figure 5A). Coiled-coil predictions (using http://toolkit.tuebingen.mpg.de/pcoils) of wild type Tea1, Tea1-5A and Tea1-5D are shown in the bottom three panels, respectively. The prediction of coiled-coil segments was made by using a 14-residue window. Red arrows indicate the regionin which an additional coiled coil appears in Tea1-5A, but not in wild type Tea1 or Tea1-5D.

Supplemental Table S1 Strain list in this study

	Genotype	
L972	h-	Fig. 1D, S1C-E
MBY1809	h- *Δcki3::ura4*$^{+}$ *leu1-32 ura4-D18*	Fig. 1D, S1C-E
TK623-5B	h- *ura4-CRIB-GFP leu1-32*	Fig. 1, 2E, 5C
TK565-4B	h- *Δcki3::ura4*$^{+}$ *ura4-CRIB-GFP ura4-D18 leu1-32*	Fig. 1, 2E, 5C
KY121	h- *tea1*$^{+}$*:5xFLAG:hph*^{r}	Fig. 2A, B, C, S5
TK499-6C	h- *Δcki3::ura4*$^{+}$ *tea1*$^{+}$*:5xFLAG:hph*^{r} *leu1-32 ura4-D18*	Fig. 2A, S5
TK622-5A	h+ *Δcki3::ura4*$^{+}$ *ura4-D18 leu1-32:cki3K44R:GFP:hphR tea1*$^{+}$*:5xFLAG:hphR his2*	Fig. 2A
TK850-7B	h- *tea1*$^{+}$*:5xFLAG:hphR Δcki3::ura4*$^{+}$ *ura4-D18 leu1-32:cki3*^{SS}*:GFP:hphR*	Fig. 2A
KY79	h- *Δcki3::ura4*$^{+}$ *ura4 leu1-32:cki3*$^{+}$*:HA:hph*^{r}*:leu1*$^{+}$	Fig. 2C
TK507-2B	h- *tea1*$^{+}$*:5xFLAG:hphR Δcki3::ura4*$^{+}$ *ura4-D18 leu1-32:cki3*$^{+}$*:HA:hphR*	Fig. 2C
TK556-4A	h- *Δcki3::ura4*$^{+}$ *ura4-D18 leu1-32:cki3*$^{+}$*:GFP:natR tea1*$^{+}$*:mCherry:kanR*	Fig. 2D
TK557-1B	h- *Δcki3::ura4*$^{+}$ *ura4-D18 leu1-32:cki3*^{K44R}*:GFP:hphR tea1*$^{+}$*:mCherry:kanR*	Fig. 2D
TK697-2A	h- *Δtea1::kanR ura4-CRIB-GFP leu1-32*	Fig. 2E
TK566-2B	h- *Δtea1::kanR Δcki3::ura4*$^{+}$ *ura4-CRIB-GFP ura4-D18 leu1-32*	Fig. 2E
TK534-1B	h- *Δcki3::ura4*$^{+}$ *ura4-D18 leu1-32:cki3*$^{+}$*:GFP:natR tea1*$^{+}$*:GBP:HIS:mcherry:kanR*	Fig. 3A, C, 5A
TK535-4B	h- *Δcki3::ura4*$^{+}$ *ura4-D18 leu1-32:cki3*^{K44R}*:GFP:hphR tea1*$^{+}$*:GBP:HIS:mcherry:kanR*	Fig. 3A, C, 5A
TK525-1	h- *tea1*$^{+}$*:GBP:HIS:mcherry:kanR leu1-32 ura4-D18*	Fig. 3B, C
TK613-1	h+ *tea4*$^{+}$*:GBP-HIS-mCherry:kanR leu1-32 ura4-D18 his2*	Fig. 3B
TK545-1	h- *Δcki3::ura4*$^{+}$ *ura4-D18 leu1-32:cki3*$^{+}$*:GBP:HIS:mcherry:hphR*	Fig. 3B
TK546-1	h- *Δcki3::ura4*$^{+}$ *ura4-D18 leu1-32:cki3*^{K44R}*:GBP:HIS:mcherry:hphR*	Fig. 3B
TK324-8B	h- *Δcki3::ura4*$^{+}$ *ura4-D18 leu1-32:cki3*$^{+}$*:GFP:natR*	Fig. 3D
KY63	h- *Δcki3::ura4*$^{+}$ *ura4-D18 leu1-32:cki3*^{K44R}*:GFP:hphR*	Fig. 3D
TK536-1C	h- *Δcki3::ura4*$^{+}$ *ura4-D18 leu1-32:cki3*$^{+}$*:GFP:natR tea1*$^{+}$*:GBP:HIS:kanR*	Fig. 3D, 5B
TK539-1B	h- *Δcki3::ura4*$^{+}$ *ura4-D18 leu1-32:cki3*^{K44R}*:GFP:hphR tea1*$^{+}$*:GBP:HIS:kanR*	Fig. 3D, 5B
TK986-5D	h- *tea1*$^{+}$*:GBP:HIS:kanR tea4*$^{+}$*:mCherry:hphR Δcki3::ura4*$^{+}$ *ura4-D18 leu1-32::cki3*$^{+}$*:GFP:natR his2*	Fig. 4A
TK989-5D	h+ *tea1*$^{+}$*:GBP:HIS:kanR tea4*$^{+}$*:mCherry:hphR Δcki3::ura4*$^{+}$ *ura4-D18 leu1-32::cki3*^{K44R}*:GFP:hphR his2*	Fig. 4A
TK637-1C	h- *tea4*$^{+}$*:5xFLAG:hphR*	Fig. 4B, 5D, S2A, S2D
TK625-12B	h- *Δcki3::ura4*$^{+}$ *ura4-D18 leu1-32:cki3*$^{+}$*:GFP:natR tea1*$^{+}$*:GBP:HIS:mcherry:kanR tea4*$^{+}$*:5xFLAG:hphR*	Fig. 4B
TK626-11A	h+ *Δcki3::ura4*$^{+}$ *ura4-D18 leu1-32:cki3*^{K44R}*:GFP:hphR tea1*$^{+}$*:GBP:HIS:mcherry:kanR tea4*$^{+}$*:5xFLAG:hphR*	Fig. 4B
TK660-2B	h- *tea4*$^{+}$*:5xFLAG:hphR tea1*$^{+}$*:GBP:HIS:mCherry:kanR*	Fig. 4B
TK626-11B	h- *Δcki3::ura4*$^{+}$ *ura4-D18 leu1-32:cki3*^{K44R}*:GFP:hphR tea4*$^{+}$*:5xFLAG:hphR*	Fig. 4B
TK631-3B	h- *Δcki3::ura4*$^{+}$ *ura4-D18 leu1-32:cki3*$^{+}$*:GFP:natR tea4*$^{+}$*:5xFLAG:hphR*	Fig. 4B, S2D
TK1006-6A	h- *Δtea4::kanR tea1*$^{+}$*:GBP:HIS:mCherry:kanR Δcki3::ura4*$^{+}$ *ura4-D18 leu1-32::cki3*$^{+}$*:GFP:natR*	Fig. 5A
TK1007-1B	h- *Δtea4::kanR tea1+:GBP:HIS:mCherry:kanR Δcki3::ura4+ ura4-D18 leu1-32::cki3*^{K44R}*:GFP:hphR*	Fig. 5A
TK1008-2C	h- *tea1*$^{+}$*:GBP:HIS:kanR tea4*$^{+}$*:GBP:HIS:mCherry:kanR Δcki3::ura4*$^{+}$ *ura4-D18 leu1-32::cki3+:GFP:natR*	Fig. 6B
TK1009-7B	h- *tea1*$^{+}$*:GBP:HIS:kanR tea4*$^{+}$*:GBP:HIS:mCherry:kanR Δcki3::ura4*$^{+}$ *ura4-D18 leu1-32::cki3*^{K44R}*:GFP:hphR*	Fig. 6B
TK703-1C	h- *tea1-5A:5xFLAG:hphR ura4-CRIB-GFP leu1-32*	Fig. 6B, C
TK749-5A	h- *Δcki3::ura4*$^{+}$ *tea1-5A:5xFLAG:hphR ura4-CRIB-GFP leu1-32 his2*	Fig. 6B
TK704-6C	h- *tea1-5D:5xFLAG:hphR ura4-CRIB-GFP leu1-32*	Fig. 6B, C
TK750-7B	h+ *Δcki3::ura4*$^{+}$ *tea1-5D:5xFLAG:hphR ura4-CRIB-GFP leu1-32 his2*	Fig. 6B
TK520-1	h- *tea1*$^{+}$*:GFP:natR leu1-32 ura4-D18*	Fig. 6D
TK965-4C	h- *tea1*$^{+}$*:GFP:natR tea4*$^{+}$*:5xFLAG:hphR leu1-32 ura4-D18*	Fig. 6D
TK984-7B	h- *Δcki3::ura4*$^{+}$ *tea1*$^{+}$*:GFP:natR tea4*$^{+}$*:5xFLAG:hphR leu1-32 ura4-D18*	Fig. 6D
TK967-1A	h- *tea1-5A:GFP:natR tea4*$^{+}$*:5xFLAG:hphR leu1-32*	Fig. 6D
TK968-9D	h- *tea1-5D:GFP:natR tea4*$^{+}$*:5xFLAG:hphR leu1-32*	Fig. 6D

	Genotype	
TK688-1C	h- cdc10-129 ura4-CRIB-GFP leu1-32	Fig. S1A
TK709-7A	h- cdc10-129 Δcki3::ura4^{+} leu1-32 ura4-CRIB-GFP	Fig. S1A
TK671-5D	h- pol1-1546 tea1^{+}:5xFLAG:hph^{r} ura4-CRIB-GFP leu1-32	Fig. S1B, S6B
TK672-2A	h+ pol1-1546 Δcki3::ura4^{+} ura4-CRIB-GFP leu1-32 his2	Fig. S1B, S6B
TK526-3B	h+ Δtea1::kan^{r} ura4-D18 leu1-32 his2	Fig. S1C, D
TK532-1C	h- Δtea1::kan^{r} Δcki3::ura4^{+} ura4-D18 leu1-32	Fig. S1C, D
TK655-5D	h- tea4^{+}:5xFLAG:hphR Δcki3::ura4+ ura4-D18 leu1-32	Fig. S2A
TK889-1	h+ tea3^{+}:5xFLAG:hphR leu1-32 ura4-D18 his2	Fig. S2A
TK900-8D	h+ Δcki3::ura4^{+} tea3^{+}:5xFLAG:hphR leu1-32 ura4-D18 his2	Fig. S2A
KY86	h- tea1^{+}:HA:kanR	Fig. S2B
TK420-4D	h- tea1^{+}:HA:kanR Δcki3::ura4^{+} ura4-D18 leu1-32	Fig. S2B
KK1	h- pom1^{+}:HA:kanR	Fig. S2B
TK262-2B	h^{-} Δcki3::ura4^{+} pom1^{+}:HA:kanR leu1-32 ura4-D18	Fig. S2B
TK830-1	h+ rga4^{+}:5xFLAG:hphR leu1-32 ura4-D18 his2	Fig. S2C
TK839-4B	h- Δcki3::ura4^{+} ura4-D18 rga4^{+}:5xFLAG:hphR leu1-32	Fig. S2C
TK831-1	h+ gef1^{+}:5xFLAG:hphR leu1-32 ura4-D18 his2	Fig. S2C
TK840-2B	h+ Δcki3::ura4^{+} ura4-D18 gef1^{+}:5xFLAG:hphR leu1-32 his2	Fig. S2C
TK997-3B	h- tea1^{+}:GFP:natR tea4^{+}:mCherry:hphR leu1-32	Fig. S3
TK621-3B	h- Δcki3::ura4^{+} ura4-D18 leu1-32:cki3^{+}:GFP:natR tea4^{+}:GBP:HIS:mcherry:kanR	Fig. S4A-C
TK620-4B	h- Δcki3::ura4^{+} ura4-D18 leu1-32:cki3^{K44R}:GFP:hphR tea4^{+}:GBP:HIS:mcherry:kanR	Fig. S4A-C
TK621-1B	h- Δcki3::ura4^{+} ura4-D18 leu1-32:cki3^{+}:GFP:natR tea4^{+}:GBP:HIS:mcherry:kanR tea1^{+}:5xFLAG:hphR	Fig. S4D
TK621-1B	h- Δcki3::ura4^{+} ura4-D18 leu1-32:cki3^{K44R}:GFP:hphR tea4^{+}:GBP:HIS:mcherry:kanR tea1^{+}:5xFLAG:hphR	Fig. S4D
TK533-3A	h- Δtea1::kanR Δcki3::ura4^{+} ura4-D18 leu1-32:cki3^{+}:GFP:natR	Fig. S4E
TK659-4C	h- Δtea1::kanR Δcki3::ura4^{+} ura4-D18 leu1-32 tea4^{+}:GBP:HIS:mcherry:kanR	Fig. S4E
TK658-11D	h- Δtea1::kan^{r} Δcki3::ura4^{+} ura4-D18 leu1-32:cki3^{K44R}:GFP:hphR tea4^{+}:GBP:HIS:mcherry:kanR	Fig. S4E
TK659-4D	h- Δtea1::kanR Δcki3::ura4^{+} ura4-D18 leu1-32:cki3^{+}:GFP:natR tea4^{+}:GBP:HIS:mcherry:kanR	Fig. S4E
TK715-5B	h- pol1-1546 tea1-5A:5xFLAG:hphR CRIB-GFP leu1-32	Fig. S6B
TK716-6B	h- pol1-1546 tea1-5D:5xFLAG:hphR CRIB-GFP leu1-32	Fig. S6B
TK785-9A	h+ pol1-1546 Δcki3::ura4^{+} tea1-5A:5xFLAG:hphR CRIB-GFP leu1-32 his2	Fig. S6B
TK786-3A	h+ pol1-1546 Δcki3::ura4^{+} tea1-5D:5xFLAG:hphR CRIB-GFP leu1-32 his2	Fig. S6B

Supplemental References

Alvarez-Tabares, I., Grallert, A., Ortiz, J.M. & Hagan, I.M. (2007). *Schizosaccharomyces pombe* protein phosphatase 1 in mitosis, endocytosis and a partnership with Wsh3/Tea4 to control polarised growth. *J. Cell Sci.* 120, 3589-3601.

Arellano, M., Niccoli, T. & Nurse, P. (2002). Tea3p is a cell end marker activating polarized growth in *Schizosaccharomyces pombe*. *Curr. Biol.* 12, 751-756.

Bähler, J. & Pringle, J.R. (1998). Pom1p, a fission yeast protein kinase that provides positional information for both polarized growth and cytokinesis. *Genes Dev.* 12, 1356-1370.

Coll, P.M., Trillo, Y., Ametzazurra, A. & Perez, P. (2003). Gef1p, a new guanine nucleotide exchange factor for Cdc42p, regulates polarity in *Schizosaccharomyces pombe*. *Mol. Biol. Cell* 14, 313-323.

Das, M., Drake, T., Wiley, D.J., Buchwald, P., Vavylonis, D. & Verde, F. (2012). Oscillatory dynamics of Cdc42 GTPase in the control of polarized growth. *Science* 337, 239-243.

Das, M., Wiley, D.J., Medina, S., Vincent, H.A., Larrea, M., Oriolo, A. & Verde, F. (2007). Regulation of cell diameter, For3p localization, and cell symmetry by fission yeast Rho-GAP Rga4p. *Mol. Biol. Cell* 18, 2090-2101.

Knippschild, U., Gocht, A., Wolff, S., Huber, N., Lohler, J. & Stoter, M. (2005). The casein kinase 1 family: participation in multiple cellular processes in eukaryotes. *Cell Signal* 17, 675-689.

Knippschild, U., Kruger, M., Richter, J., Xu, P., Garcia-Reyes, B., Peifer, C., Halekotte, J., Bakulev, V. & Bischof, J. (2014). The CK1 family: contribution to cellular stress response and its role in carcinogenesis. *Frontiers in oncology* 4, 96.

Martin, S.G., McDonald, W.H., Yates, J.R., 3rd & Chang, F. (2005). Tea4p links microtubule plus ends with the formin For3p in the establishment of cell polarity. *Dev. Cell* 8, 479-491.

Mata, J. & Nurse, P. (1997). Tea1 and the microtubular cytoskeleton are important for generating global spatial order within the fission yeast cell. *Cell* 89, 939-949.

Niccoli, T., Arellano, M. & Nurse, P. (2003). Role of Tea1p, Tea3p and Pom1p in the determination of cell ends in *Schizosaccharomyces pombe*. *Yeast* 20, 1349-1358.
